# Revisiting the nexus between fiscal decentralization and CO_2_ emissions in South Africa: fresh policy insights

**DOI:** 10.1186/s40854-023-00453-x

**Published:** 2023-02-02

**Authors:** Maxwell Chukwudi Udeagha, Marthinus Christoffel Breitenbach

**Affiliations:** Department of Economics, School of Economics, University of Pretoria, Hatfield Campus, Private Bag X20, Hatfield, 0028 South Africa

**Keywords:** Fiscal decentralization, Trade openness, CO_2_ emissions, Dynamic ARDL simulations, Energy consumption, EKC, Cointegration, Economic growth, Industrial value-added, South Africa, F18, F13, Q56, O13, F1, F41

## Abstract

The argument over fiscal decentralization and carbon dioxide emission (CO_2_) reduction has received much attention. However, evidence to back this claim is limited. Economic theory predicts that fiscal decentralization affects environmental quality, but the specifics of this relationship are still up for debate. Some scholars noted that fiscal decentralization might lead to a race to the top, whereas others contended that it would result in a race to the bottom. In light of the current debates in environmental and development economics, this study aims to provide insight into how this relationship may function in South Africa from 1960 to 2020. In contrast to the existing research, the present study uses a novel dynamic autoregressive distributed lag simulation approach to assess the positive and negative changes in fiscal decentralization, scale effect, technique effect, technological innovation, foreign direct investment, energy consumption, industrial growth, and trade openness on CO_2_ emissions. The following are the main findings: (i) Fiscal decentralization had a CO_2_ emission reduction impact in the short and long run, highlighting the presence of the race to the top approach. (ii) Economic growth (as represented by the scale effect) eroded ecological integrity. However, its square (as expressed by technique effect) aided in strengthening ecological protection, validating the environmental Kuznets curve hypothesis. (iii) CO_2_ emissions were driven by energy utilization, trade openness, industrial value-added, and foreign direct investment, whereas technological innovation boosted ecological integrity. Findings suggest that further fiscal decentralization should be undertaken through further devolution of power to local entities, particularly regarding environmental policy issues, to maintain South Africa’s ecological sustainability. South Africa should also establish policies to improve environmental sustainability by strengthening a lower layer of government and clarifying responsibilities at the national and local levels to fulfill the energy-saving functions of fiscal expenditures.

## Introduction

Although the world has been battling the COVID-19 pandemic for more than a year and a half, climate change is still at the top of the international intervention roadmap (Udeagha and Ngepah 2022a, 2022b). The potential effects of global warming are globally acknowledged, such as increasing temperatures that threaten ecosystems and people. These hazards are categorized as transitional threats and biophysical problems (Xia et al. 2021; Yang et al. 2021a, b; Wang et al. 2021). Emissions of greenhouse gases (GHGs) from commercial endeavors are one of the most important links between the economic sector and global warming. Governments have taken several steps to mitigate climate change throughout the years, and GHG emissions have been a major area of study for many researchers. Scholars’ recently focused on the issue of growing GHG emissions, particularly carbon emissions (CO_2_), as a major cause of environmental damage. Nevertheless, one of the most serious global challenges for authorities and policy decisions in the twenty-first century is global warming (Cheng et al. 2021; Tufail et al. 2021). Although climate change is a significant issue, different regions of the world experience its effects differently and have various obligations to cut emissions, as outlined in the Paris Agreement (UNEP 2018; IPCC 2019).

Researchers and policymakers are now focusing on the causes of environmental problems to boost sustainable production and consumption owing to the degradation of ecological and natural resources (Xin et al. 2022; Khan et al. 2022a, b; Jahanger et al. 2022). The idea of a clean atmosphere has acquired momentum on a global scale. Every country’s economy and people’s quality of life are affected by extreme weather conditions and other issues brought on by rising temperatures (Ahmed et al. 2022; Chishti et al. 2022). However, national ecological responses have remained constant based on the indicators of socioeconomic development, monetary policy, trade openness, usage of fossil fuels, and several other significant factors. Additionally, the accelerated industrial growth, infrastructural expansion, and increased commercial operation that have stemmed from expanding globalization have increased energy demand (Shakib et al. 2022). Thus, GHG emissions have kept growing. The primary cause of climate change is CO_2_ emissions from the combustion of fossil fuels (Xue et al. 2021; Murshed 2021, 2020). Global carbon emissions are one of the key reasons for the deteriorating environmental quality. Reports showed that atmospheric CO_2_ concentration has increased by 45% in the past 130 years (Murshed et al. 2021a, b).

Environmental degradation affects every country, making it a growing global problem. Industrialized countries, such as China, Germany, Japan, Russia, India, and the United States, are accountable for protecting the environment as significant GHG polluters (World Bank 2021). In addition, a collaboration between these countries and a few others is necessary to achieve the aim of reducing global CO_2_ emissions. However, limiting CO_2_ emissions would result in lower output, which would impede economic growth. The reason is that this case is linked to energy usage, which is crucial for economic development (Jain et al. 2021; Li et al. 2021a, b, c; Rani 2021). Thus, following or implementing measures that are intended to curb rising temperatures directly is exceedingly difficult for these nations. Therefore, more effective methods for promoting sustainable economic growth and better environmental circumstances are required. Some authorities worldwide have employed a variety of measures in their efforts to tackle environmental destruction and extreme weather events (Z. Khan et al. 2021b, a; Lin and Zhou 2021). One of these tactics regarded as an effective strategy to improve environmental health is fiscal decentralization.

Fiscal decentralization is a comprehensive system, which comprises a framework for transferring control of revenue, spending, and the associated liabilities to a lower level of government (Xiao-Sheng et al. 2021; Xu 2022; Du and Sun 2021). Decentralization of fiscal revenue and spending is included. In many countries globally, fiscal decentralization has emerged as one of the most crucial policy tools for local economic and social progress. The reason is that it encourages local governments to “race to the top” by enforcing better environmental standards owing to the NIMBYism effect (Ji et al. 2021; Yang et al. 2021a, b; Guo et al. 2020). That is, local governments have a better understanding of residents’ demand preferences and the current conditions of regional environmental degradation than central governments. Thus, local governments are better positioned to set higher environmental standards to improve the environmental quality in the affected regions. Indeed, in recent years, the globe has seen a considerable increase in central governments’ tendency to delegate their environmental policy development and implementation responsibilities to local governments. This event also allows local governments to increase resource allocation efficiency (Xiao-Sheng et al. 2021). In addition, ample evidence shows that fiscal decentralization addresses the effects of poor service delivery and poor performance of central governments under centralized systems that foster a rent-seeking culture and tolerate competencies and failures to meet public demand preferences in many less developed countries (Yang et al. 2021a, b).

South Africa is among the few nations to have successfully implemented comprehensive policy measures to decentralize its fiscal system by assigning and distributing functions to different layers of authority to improve service delivery and resource allocation efficiency (Gumede et al. 2019; Amusa and Mabugu 2016). The country’s 1996 constitution divides the government into three tiers (layers) that are interconnected, interdependent, and distinct: national (central), nine province (regional), and 284 municipal administrations, each with its own set of tasks, functions, and authorities. The national government oversees the country’s affairs and shares responsibility with provincial (regional) governments. Meanwhile, provincial governments are responsible for providing fundamental services, such as welfare, education, and health care to improve people’s standard of living. The provision of power, sanitation, and water is the responsibility of the local administration. South Africa’s fiscal decentralization comprises the distribution of expenditure and revenue-related responsibilities to provincial governments. The above details make South Africa an interesting scenario research for analyzing how fiscal decentralization has improved the nation’s environmental quality.

With approximately 390 million tonnes of CO_2_ emissions in 2020, South Africa is believed to be the biggest contributor to climate change in Africa and the 15th largest CO_2_ producer in the world (1.09% of emissions worldwide) (World Bank 2021). The nation’s steadily rising CO_2_ emissions seem to be predominantly caused by coal burning (Udeagha and Ngepah 2022c). The major element of CO_2_ emissions in South Africa comes from coal, which is also the country’s dominant source of energy. Approximately 77% of all energy production is provided by coal, which is used for electricity production (53%), iron and steel enterprises (12%), chemical plants (33%), and household heating and cooking purposes (Udeagha and Breitenbach 2021). Thus, South Africa is a perfect destination to study how fiscal decentralization affects the nation’s CO_2_ emissions.

Few empirical research studied the impacts of fiscal decentralization on economic growth in South Africa. For example, Gumede et al. (2019) investigated the role of fiscal decentralization in promoting local self-government. They found that effective fiscal decentralization is a tool that facilitates greater efficiency and effectiveness in the local government while promoting economic growth in South Africa. According to Albehadili and Hai (2018), fiscal decentralization might be a solution to the issues that central and local governments confront. The reason is that fiscal decentralization boosts economic efficiency and makes it easier for the government to respond to the demands of its inhabitants. Moche et al. (2014) used a panel vector autoregression and found that fiscal decentralization reduces poverty and improves economic performance in all eight metropolitan municipalities. Better special-purpose policies have been implemented through fiscal decentralization to ensure that specified services are given to reduce poverty and boost economic growth in South Africa. Amusa and Mabugu (2016) studied the fiscal decentralization–regional inequality link. They discovered that fiscal decentralization improves responsiveness and increases the overall efficiency of the public sector’s in-service delivery, thereby enhancing economic development and reducing regional disparities. Bikam et al. (2015) investigated the influence of fiscal decentralization in tackling water and sanitation infrastructure backlogs in South Africa using the Municipal Infrastructure Grant (MIG) framework in Mahikeng and Thulamela Local Municipalities. The findings revealed that fiscal decentralization improves the capacity of under-resourced municipalities in South Africa’s Mahikeng and Thulamela Local Municipalities. Thus, the MIG system can be used to solve difficulties of water and sanitation infrastructure backlogs.

Some studies regarded the relationship between fiscal decentralization and CO_2_ emissions in a global context, such as Li et al. (2021a, b, c) for Pakistan; Lin and Zhou (2021), Cheng et al. (2020b, 2021), and Chen and Liu (2020) for China; Khan et al. (2021b, a), Tufail et al. (2021), Su et al. (2021), and Shan et al. (2021) for Organisation for Economic Co-operation and Development (OECD)^1^ countries; Wang et al. (2021) and Ji et al. (2021) for fiscally decentralized economies; and Jain et al. (2021) for Asian economies.

Although previous works on the role of fiscal decentralization in influencing environmental quality have progressed, several crucial areas are left unexplored. The present study incorporates such elements to contribute meaningfully to the growing body of literature. First, no research has been done in South Africa to analyze the dynamic relationship between fiscal decentralization and environmental quality and provide insight into the precise processes by which this link may operate. Second, no earlier research has examined the relationship between fiscal decentralization and ecological sustainability using a sophisticated estimating technique, such as the dynamic autoregressive distributed lag (ARDL) simulation approach of Jordan and Philips (2018). Third, none of the previous research appears to have used the frequency domain causality (FDC) test of Breitung and Candelon (2006), the most effective and efficient testing paradigm. Thus, using the FDC framework in this current research enables proper accounting of persistent causation among variables over long, short, and medium timescales. Fourth, the previous research ignored the second-generation econometric approaches to examine the effects of structural breaks.

Considering the aforementioned knowledge gaps, this study mainly aims to explore the dynamic association between fiscal decentralization and environmental quality in South Africa from 1960 to 2020. The current study makes several contributions to the body of literature. First, from a theoretical perspective, the study primarily contributes by investigating the existence of the race to the top theory. This case is frequently attributed to a decentralized fiscal structure that enables local authorities to supervise environmentally harmful businesses and thereby shift them outside of their jurisdiction. The fundamental reason is that regional rivalry may have a “race to the top” effect, resulting in tighter environmental rules at greater degrees of fiscal decentralization, which makes any fiscal decentralization beneficial for the environment. In summary, the existing body of literature has already produced several useful findings (Lingyan et al. 2022; Sun et al. 2022). Although these studies are well supported by references and serve as an inspiration to us, there is still potential for development. The following are the main issues: the underlying causes of fiscal decentralization’s consequences on environmental pollution, or, to put it another way, if fiscal decentralization might cause environmental “race to the top” behavior among governments. The literature currently available is only at the qualitative debate level (He et al. 2022; Zhang et al. 2022) and does not provide a definitive solution and even lacks robust quantitative evidence. Hence, the analysis should undergo additional empirical testing. We dynamically explore the effect of fiscal decentralization on CO_2_ emissions in this study using the standard environmental Kuznets curve (EKC) methodology to further confirm whether fiscal decentralization causes governments to engage in an environmental “race to the top.” Second, the study makes an important contribution to scholarship from methodological and empirical viewpoints by using a sophisticated estimation procedure, the novel dynamic ARDL simulation methodology. This procedure is designed to improve the shortcomings of the previous ARDL model to evaluate different model characteristics in the short and long run. The novel dynamic simulated ARDL model aims to simulate, estimate, and dynamically prepare predictions of counterfactual changes in a single regressor and its impact on regressions while keeping the other independent variables constant (Jordan and Philips 2018). This special framework can continuously compute, reproduce, and explore the graphs and the short- and long-term connections between positive and negative variables. Pesaran et al. (2001) developed the basic ARDL, but it can only evaluate long- and short-term linkages among variables. The study’s parameters are all stationary and have a mixed integration order of I(0) and I(1), allowing the use of a single dynamically simulated ARDL model. The variables investigated in this research are timely enough to fulfill the criteria for creating a novel dynamic simulation ARDL model. Using this framework permits to reveal the counterfeit alterations in regressors and their impacts on regression. The novel dynamic ARDL error correction equation is derived from earlier works (Jordan and Philips 2018; Udeagha and Muchapondwa 2022a). Third, this work further adds to scholarship on the methodological front by employing a FDC approach for South Africa’s data to assess the effect of fiscal decentralization on environmental sustainability using three frequencies: ωi = 0.05, 1.50, and 2.50 to evaluate the causal linkage for short, medium, and long run, respectively. This work further advances scholarly work on the methodological front using this robust strategy, allowing us to estimate the causal relationship among variables for the short, medium, and long run. This strategy is the most effective and efficient testing framework for appropriate accounting of persistent causation among variables across long, short, and medium timeframes. Moreover, this research uses the FDC approach as a robustness exercise. Fourth, the structural break unit root test developed by Narayan and Popp (2010) is used to evaluate the study series’ stationarity characteristics. The conceivably damaging implications of structural breaks in the variables are uncovered using this approach, known as the multiple structural break procedure. Empirical evidence suggests that structural breaks are chronic and have an impact on a variety of economic determinants, particularly indices for fiscal decentralization and CO_2_ emissions. Thus, failure to adjust for structural breaks could produce incorrect and inconsistent conclusions. This study additionally tests for cointegration in the presence of endogenous structural breaks using the Gregory–Hansen test of cointegration with regime transitions. Finally, this study contributes to the body of literature on the relationship between fiscal decentralization and ecological sustainability in the presence of trade openness. That is, this study thoughtfully leverages the novel and ground-breaking trade openness proxy of Squalli and Wilson (2011) to account for two dimensions of trade openness: trade share in the gross domestic product (GDP) and trade size relative to global trade. We differ from other research that evaluated and generally characterized trade openness utilizing usual trade intensity (TI) by employing the Squalli and Wilson metric.

The rest of the paper is organized as follows. Sect. "Literature review and contributions of the study" provides a review of the literature on the fiscal decentralization–CO_2_ emission nexus. Sect. "Material and methods" deals with the material and research strategy methodology, and Sect. "Empirical results and their discussion" presents the results. Finally, Sect. "Conclusions and policy implications" concludes with policy implications.

## Literature review and contributions of the study

This section has three parts. The first section discusses the theoretical basis for the relationship between fiscal decentralization and environmental quality. The second section covers this relationship. Finally, the last section compiles the literature gaps.

### Theoretical underpinning

The effects of fiscal decentralization on the environment have been well studied theoretically. On the one hand, supporters of the “race to the top theory,” such as Glazer (1999), Wilson (1996), and Markusen et al. (1993), contend that greater fiscal decentralization is associated with improved environmental health. The idea of a race to the top is connected to a decentralized fiscal framework that enables local governments to monitor ecologically harmful firms and push them outside their purview. The local government can instigate a race to the top rivalry among local firms because they have the knowledge advantage to effectively address local concerns, given that different places have unique local difficulties. According to the campaigners of the “race to the bottom theory,” fiscal decentralization lowers ecological integrity because governments are incentivized to pass overly lax environmental regulations to draw in mobile capital (Woods 2006; Potoski 2001; Levinson 2003; Engel 1996). In a “race to the bottom,” when governments are pressured to loosen environmental regulations to retain their commercial and investment prospects against states with more “investment-friendly” laws, there is competition for industrial expansion.

### Fiscal decentralization–environmental quality nexus

The environmental impacts of fiscal decentralization have attracted prominence since Tiebout (1956) proposed the “vote with the feet” theory. Therefore, several researchers have begun to look into how fiscal decentralization affects CO_2_ emissions. The literature has recently given more emphasis to the effect of fiscal decentralization on CO_2_ emissions. However, evidence for their connection is conflicting and may be divided into two strands.

On the one hand, the literature contended that a higher level of fiscal decentralization, or the “race to the top,” is more effective in reducing CO_2_ emissions and achieving pollution control.

From this viewpoint, fiscal decentralization not only aids local governments in improving resource allocation utilization, understanding the level of regional environmental damage, and prioritizing occupant demands (Millimet 2003; Mu 2018) but also sparks a “race to the top” among local governments by encouraging them to strengthen environmental requirements (Levinson 2003). The common consensus is that local governments should promote economic development through fiscal decentralization while enhancing environmental quality (Yang et al. 2020). For instance, Hao et al. (2020) reported the same outcomes for the relationship between emissions and fiscal decentralization, demonstrating that fiscal decentralization has a beneficial impact on CO_2_ emissions. Similarly, Xu (2022) studied the relationship between fiscal decentralization and environmental management efficiency in China. They discovered that fiscal decentralization considerably improves environmental governance and efficiency, reducing the rate of environmental degradation in the country. Tufail et al. (2021) used panel data from seven highly fiscally decentralized OECD countries to investigate the effect of fiscal decentralization on CO_2_ emissions and observed that fiscal decentralization improves the environment for the OECD group. Similarly, Rani (2021) utilized the novel Method of Moments Quantile Regression approach and showed that fiscal decentralization helped to minimize environmental pollution in Asian nations from 1998 to 2018. In addition, Jain et al. (2021) explored the dynamic influence of fiscal decentralization on environmental quality for nine Asian nations from 1984 to 2017. They found that fiscal decentralization reduces CO_2_ emissions while having an unbalanced influence on environmental quality. Lingyan et al. (2022) found a similar result when they looked at the nonlinear impact. They further observed that fiscal decentralization helps to regenerate the environment by utilizing a variety of tactics and institutions that help to restore environmental quality. Fiscal decentralization helps to reduce environmental degradation and ensures environmental sustainability by establishing strong environmental standards. Lin and Zhou (2021) looked at the influence of fiscal decentralization on ecological sustainability and energy efficiency from the standpoint of vertical fiscal imbalance. They noticed that a vertical fiscal imbalance is caused by a mismatch of spending and revenue decentralization, which has a significant influence on ecological integrity and energy efficiency. Furthermore, the impact is unevenly distributed across the country, such that it worsens ecological integrity and energy efficiency in the rich and eastern zones but improves ecological sustainability and energy efficiency in the poor, western, and central provinces as these zones gain additional fiscal transfers. Li et al. (2021a, b, c) studied the asymmetric influence of fiscal decentralization on environmental quality in Pakistan and revealed that fiscal decentralization promotes environmental quality. Fiscal decentralization improves the effectiveness of the public sector by providing better information and perfect knowledge to local authorities, resulting in higher living standards, long-term economic development, and lower CO_2_ emissions. Cheng et al. (2021) evaluated the role of fiscal decentralization and technological innovation (TECH) on ecological quality in the context of globalization and GDP in China over the period 2005Q1 to 2018Q4. Their results showed that fiscal decentralization and TECH are important variables in enhancing ecological sustainability, whereas globalization and GDP deteriorate ecological quality. Khan et al. (2021a, 2021b) conducted an empirical study on the environmental impact of fiscal decentralization, considering the roles of human capital and institutions. They found that fiscal decentralization enhances environmental quality in seven OECD economies from 1990 to 2018. The authors further demonstrated that when human capital and institutional quality are enhanced, fiscal decentralization improves environmental quality dramatically. Cheng et al. (2020b) assessed the nonlinear effect of fiscal decentralization on ecological sustainability in China using a dynamic panel data methodology and the logarithmic mean Divisia index decomposition approach. Xia et al. (2022a) posited that fiscal decentralization in eastern China had made a significant contribution to the performance of environmental governance. They used panel data from 31 provinces from 2010 to 2019 to compare the influence on regional carbon emissions. Similarly, Xia et al. (2022b) used the first-order differential dynamic panel econometrics model to examine the effects of fiscal decentralization reform on carbon dioxide emissions in China from 2010 to 2019. They noted that fiscal imbalance decreased CO_2_ emissions as a result of the decentralization of revenue, whereas expenditure asymmetry eroded CO_2_ emission control.

By contrast, the second body of scholarship contends that fiscal decentralization has caused environmental deterioration, leading to a “race to the bottom” (Liu et al. 2019). Many researchers agreed with this viewpoint and believed that local governments are more likely to participate in a “race to the bottom.” This case entails reducing environmental protections to provide more room for economic growth and, consequently, encourage a rise in CO_2_ emissions. For instance, Zhang et al. (2017) investigated the effect of fiscal decentralization on the operational modalities of Chinese pollution control while adjusting for the spatial correlations of CO_2_ emissions. They suggested that the Chinese approach to decentralization generates a system that noticeably fosters CO_2_ emissions, resulting in the “green paradox.” Zhang et al. (2016) also corroborated similar findings. Xiao-Sheng et al. (2021) recently examined the environmental effects of fiscal decentralization in 270 Chinese cities from 2007 to 2016 and showed that the quality of the environment in Chinese cities declined because of fiscal decentralization. Similarly, Chen and Liu (2020) examined the impact of fiscal decentralization on carbon emissions in 31 Chinese provinces from 2003 to 2017 using the geographic Durbin framework. They identified fiscal decentralization as a major factor contributing to environmental deterioration from the perspectives of fiscal spending and fiscal revenue devolution. Similarly, Xia et al. (2021) demonstrated that fiscal decentralization greatly relates to increased CO_2_ emissions inside the area and in its surroundings, using a panel data set of 30 provinces and regions in Mainland China. Furthermore, Lin and Zhou (2021) observed that fiscal decentralization had a negative impact on ecological sustainability in China’s economically industrialized and eastern areas. Utilizing the dynamic spatial Durbin framework, Yang et al. (2021a, b) derived similar conclusions. Their findings revealed that fiscal decentralization and urban sprawl exacerbate ecological degradation. In addition, Zhan et al. (2022) measured and evaluated green total factor productivity using panel data from 30 Chinese provinces and cities between 2012 and 2018. They noted that the decentralization of fiscal revenue and expenditure significantly stifles the growth of green total factor productivity at the provincial level. The authors also found that in central and western regions with regional variation, fiscal decentralization reduces the productivity of the green total factor. Table 1 offers a review of research on the connection between fiscal decentralization and ecological sustainability to provide additional assessments among countries worldwide.

**Table 1:** Synopsis of studies

S/N	Investigator (s)	Timeframe	Nation (s)	Technique(s)	Findings
1	Cheng and Zhu (2021)	2003–2016	China	Dynamic spatial Durbin model	Fiscal decentralization triggers CO_2_ emissions
2	Cheng et al. (2021)	2005–2018	China	DOLS, CCR, FMOLS	Fiscal decentralization reduces the level of emissions
3	Du and Sun (2021)	2003–2018	Chinese provinces	PSTR	Fiscal decentralization deteriorates environmental quality
4	Xiao-Sheng et al. (2021)	2007–2016	270 Chinese cities	Spatial regression methods	Fiscal decentralization impedes environmental quality
5	Xia et al. (2022a, b)	2006–2016	30 Chinese provinces	Spatial Durbin model	Fiscal decentralization increases carbon emissions
6	Yang et al. (2021a, b)	2004–2018	269 Chinese cities	Dynamic spatial Durbin model	Fiscal decentralization accelerates environmental quality
7	Xu (2022)	1995–2017	China	ARDL model	Fiscal decentralization improves environmental quality
8	Wang et al. (2021)	1984–2019	Fiscally decentralized economies	Quantile regression model, FMOLS	Fiscal decentralization decreases carbon emissions
9	Tufail et al. (2021)	1990–2018	7 OECD	CS-ARDL, AMG	Fiscal decentralization reduces carbon emissions
10	Rani (2021)	1998–2018	48 countries	MMQR, FMOLS, DOLS, FE-OLS	Fiscal decentralization improves environmental quality
11	Phan et al. (2021)	1984–2017	9 Asian economies	Dynamic panel ARDL model	Fiscal decentralization improves environmental quality
12	Lingyan et al. (2022)	1990–2019	Top 10 highly decentralized countries	MMQR	Fiscal decentralization improves environmental quality
13	Lin and Zhou (2021)	2000–2017	Chinese provinces	Fixed-effect model	Fiscal decentralization improves environmental quality
14	Li et al. (2021a, b, c)	1984–2018	Pakistan	Asymmetric ARDL model	Fiscal decentralization improves environmental quality
15	Khan et al. (2021b, a)	1990–2018	7 OECD economies	CS-ARDL	Fiscal decentralization improves environmental quality
16	Du and Sun (2021)	2003–2018	285 Chinese prefecture-level cities	PSTR	Fiscal decentralization deteriorates environmental quality
17	Su et al. (2021)	1990–2018	OECD economies	CS-ARDL	Fiscal decentralization improves environmental quality
18	Cheng et al. (2021)	2005–2018	China	DOLS, FMOLS, CCR	Fiscal decentralization reduces carbon emissions
19	Cheng et al. (2020a)	1997–2016	30 Chinese provinces	LMDI framework	Fiscal decentralization deteriorates environmental quality
20	Cheng et al. (2020b)	1997–2015	China	LMDI, dynamic panel regression model	Fiscal decentralization reduces carbon emissions
21	Chen and Liu (2020)	2003–2017	31 Chinese provinces	Spatial Durbin model	Fiscal decentralization deteriorates environmental quality
22	Chen and Chang (2020)	2003–2017	30 Chinese provinces	Spatial economic model	Fiscal decentralization reduces the level of emissions
23	Zhou and Zhang (2020)	2007–2017	30 Chinese provincial-level administrative divisions	SLM	Fiscal decentralization reduces the level of emissions
24	Wen and Lee (2020)	2003–2011	China	DID method	Fiscal decentralization reduces the level of emissions
25	Li et al. (2021b)	1984–2018	Pakistan	NARDL	Fiscal decentralization improves environmental quality
26	Hao et al. (2020)	1995–2015	China	Two-equation regression model	Fiscal decentralization decreases carbon emissions
27	Ji et al. (2021)	1990–2018	Switzerland, Spain, Germany, Canada, Belgium, Austria, and Australia	Panel data econometric tools	Fiscal decentralization escalates carbon emissions
28	Guo et al. (2020)	2009–2011	Chinese provinces	Panel data econometric tools	Fiscal decentralization escalates carbon emissions
29	Yang et al. (2020)	2003–2016	278 Chinese cities	Fixed-effect model and quantile regression	Fiscal decentralization worsens environmental quality
30	Chen and Liu (2020)	1996–2016	China	LMDI method	Fiscal decentralization increases environmental degradation
31	Yang et al. (2020)	2005–2016	China	Spatial Durbin model	Fiscal decentralization improves environmental quality
32	Khan et al. (2020a, b)	1990–2018	7 OECD countries	ARDL approach	Fiscal decentralization improves environmental quality
33	Ahmad et al. (2020)	2003–2016	10 top Chinese cities	GMM	Fiscal decentralization improves environmental quality
34	Kuai et al. (2019)	1990–2016	30 Chinese provinces	Spatial autoregression model	Fiscal decentralization intensifies level of emissions
35	Yang et al. (2019)	2005–2016	China	SDM	Fiscal decentralization worsens environmental quality
36	Liu et al. (2019)	2000–2012	30 Chinese municipalities and provinces	Hamilton function method, fixed-effect method, threshold regression	Fiscal decentralization intensifies level of emissions
37	Kuai et al. (2019)	1998–2016	31 Chinese provinces	Spatial Durbin model	Fiscal decentralization improves environmental quality
38	Mugableh (2019)	1978–2017	Jordan	ARDL and VECM	Fiscal decentralization improves environmental quality
39	Zhang et al. (2017)	1995–2012	29 Chinese provinces	GMM	Fiscal decentralization deteriorates environmental quality
40	Hector (2017)	2003–2015	Chinese low- and high-income provinces	Panel OLS	Fiscal decentralization worsens environmental quality
41	Assuncao and Schutze (2017)	1970–2006	Brazil	Prediction method	Fiscal decentralization improves environmental quality
42	He (2015)	1995–2010	Chinese provinces	System GMM	Fiscal decentralization worsens environmental quality
43	Li et al. (2013)	1991–2001	China	SBM model	Fiscal decentralization deteriorates environmental quality
44	Kim (2011)	2004–2008	South Korea	Panel analysis	Fiscal decentralization deteriorates environmental quality
45	Sigman (2007)	1979–1999	35 selected countries	Weighted least squares	Fiscal decentralization has no effect on carbon emissions
46	Konisky (2007)	1985–2000	USA	Strategic interaction models	Fiscal decentralization increases the level of emissions
47	Millimet (2003)	1920–2000	USA	Multivariate analyses of the state policy	Fiscal decentralization decreases the level of emissions

*DOLS* Dynamic ordinary least squares; *FMOLS* Fully modified ordinary least squares; *PSTR* Panel smooth transition regression; *CCR*: Canonical cointegrating regression; *PSTR* Panel smooth transition regression; *LMDI* Logarithmic mean Divisia index decomposition model; *SLM* Spatial lag model; *SDM* Spatial Durbin model; *DID* Difference-in-difference method; *ARDL* Autoregressive distributed lag model; *AMG* Augmented mean group; *MMQR* Method of moments quantile regression; *FE-OLS* Fixed effect-ordinary least squares; *NARDL* Non-linear autoregressive distributed lag model; *GMM* Generalized method of moment framework; *VECM* Vector error correction model; *OECD* Organisation for Economic Co-operation and Development; *CS-ARDL* Cross-sectional augmented autoregressive distributed lag

### Summarizing literature gaps

Earlier research on the influence of fiscal decentralization on environmental quality has advanced. However, it has also left several important questions unanswered. These components are included in the current study to significantly add to the expanding body of knowledge. First, no study in South Africa examines the dynamic relationship between fiscal decentralization and ecological sustainability and offers insight into how this relationship may function. Second, none of the preceding studies used a sophisticated estimation method, such as the dynamic ARDL simulations methodology used by Jordan and Philips (2018), to explore the relationship between fiscal decentralization and environmental integrity. Third, none of the previous research appears to have used the FDC test of Breitung and Candelon (2006), the most effective and efficient testing paradigm. Therefore, using the FDC framework in this current research enables proper accounting of persistent causation among variables over long, short, and medium timescales. Fourth, the previous research has ignored the second-generation econometric approaches to examine the effects of structural breaks.

## Material and methods

This study examines the long- and short-run coefficients of the variables using the innovative, dynamic ARDL simulation framework. The research employs the FDC methodology of Breitung and Candelon (2006) to account for permanent causation over long, short, and medium periods among the variables studied. In this work, the FDC framework is also employed to verify robustness.

### Functional form

This research investigates the fiscal decentralization–CO_2_ emissions nexus for South Africa by using the traditional EKC hypothesis, the robust empirical method employed in earlier studies. The study used the traditional EKC framework for the following reasons: (i) as ecological degradation rises with the production processes, the EKC framework has gained considerable importance recently. However, emissions begin to drop beyond a certain level for the following grounds (Lieb 2003): stringent pollution regulations are activated because of concerns of irreversible disaster, expanding the market for an improved ecological quality; one source of GHG emissions is substituted for the other, resulting in a reduction in emissions; advances in technology allow production to rise while emitting fewer carbon emissions. (ii) The EKC framework poses crucial scientific questions on how macroeconomic variables, such as trade and growth, can cause global warming, including launching a major research initiative (Copeland and Taylor 2004). (iii) Credible proof shows that an income effect improves the sustainability of the environment (Copeland and Taylor 2005). Furthermore, significant signs show that this income effect operates because environmental legislation becomes more stringent as per capita income rises. Thus, endogenous policy responses should be considered when examining the environmental consequences of macroeconomic factors. Prior studies have also proposed and employed alternative environmental models, such as IPACT, ImPACT, and STIRPAT. For instance, York et al. (2003) investigated the relationship between the variables that cause CO_2_ emissions using three analytical techniques (IPAT, ImPACT, and STIRPAT).^2^ They argued that the IPAT and ImPACT frameworks are ineffective for testing hypotheses and cannot be used to analyze the impact of separate elements on the ecological footprint because the components are multiplicatively interrelated. From another aspect, the STIRPAT framework aids studies in determining the amount to which certain human activities have environmental impacts (Liu et al. 2015a, b). The framework calculates the net environmental impact of each polluting element and allows hypothesis testing (York et al. (2003). The STIRPAT approach is not an accounting equation. However, this approach is a valuable analytical method for understanding the interaction between variables and the ecological footprint (Anser et al. 2020).

The EKC hypothesis holds that environmental damage gets worse as income increases, particularly in the beginning phases of a profound transformation. This happens because minimizing ecological damage is less important to society than obtaining faster economic growth. Therefore, an increase in income leads to a boost in climate change. Contextually and profoundly, this idea explicates the positive relationship between the scale effect (determined by income) and CO_2_ emissions. From another aspect, environmental deterioration is more severe during the industrial stage of development and gets worse as the economic system grows increasingly industrialized and moves away from productive activities dominated by agriculture. People start to care more about the environment, which leads to the adoption of stronger environmental regulations to improve ecological integrity. Therefore, during the modern industrialized phase of social transformation, the yearning for environmental conservation and the state’s enforcement of more stringent environmental regulations significantly improved ecological integrity. Therefore, as income (economic growth) increases, environmental deterioration declines, and this idea is intrinsically supported by the negative connection between the two variables.

We therefore present the standard EKC hypothesis following Udeagha and Ngepah (2021a, b) as follows:

1 $${\text{CO}}_{2t} = F\left( {SE, \;TE} \right),$$

where CO_2_ denotes CO_2_ emissions, a measure of the environment; SE represents scale effect denoting income; and TE stands for technique effect (TE) capturing income squared. When log-linearized, Eq. (1) gives the following:

2 $${\text{InCO}}_{2t} = \alpha + \varphi InSE_{t} + \beta InTE_{t} + \varepsilon_{t} .$$

The following conditions must be met for the EKC hypothesis to be justified:$\varphi > 0$ and $\beta < 0$. Economic expansion has a scale impact (SE) that causes global warming to escalate as income goes up. However, as environmental regulations get stricter, the technique’s influence reduces ecological damage. We argue for Eq. (2) by adding the environmental consequences of fiscal decentralization and some important control variables as follows:

3 $$\begin{aligned} {\text{ InCO}}_{2t} & = \alpha + \varphi InSE_{t} + \beta InTE_{t} + \psi InFISD_{t} + \rho InTECH_{t} + \pi InEC_{t} \\ & \quad + \delta InFDI_{t} + \tau InOPEN_{t} + \omega InIGDP_{t} + U_{t,} \\ \end{aligned}$$

where $In\;FISD_{t}$ denotes fiscal decentralization; $In\;IGDP_{t}$ stands for industrial value-added; $In\;OPEN_{t}$ signifies trade openness; $In\;FDI_{t}$ denotes foreign direct investment (FDI); $In\;EC_{t}$ signifies energy consumption (EC); $In\;TECH_{t}$ is TECH; and all variables are in their natural log. $\varphi ,\beta ,\psi ,\rho , \pi , \delta \tau ,\;{\text{and}}\; \omega$ are the estimable coefficients, which capture various elasticities, and $U_{t}$ is the stochastic error term, including its standard features.

### Measuring trade openness

The shortcomings of earlier TI methodologies are successfully addressed in this research using the composite trade intensity (CTI) model proposed by Squalli and Wilson (2011). The CTI effectively considered two aspects of trade openness—the share of trade in GDP and the volume of trade relative to global trade. Our study differs from earlier ones that employed it as a measure and proxy for trade openness using the Squalli and Wilson measure of trade openness instead of the conventional TI. Furthermore, the limitations of the conventional TI are successfully overcome by using this thorough approach to quantifying trade openness. The innovative CTI essentially includes more crucial information on a country’s trade participation quota to the international economy (Squalli and Wilson 2011). Additionally, this unique proxy for trade openness mirrors the trade outcome reality as it considers two dimensions of a country’s interactions with the rest of the world. We express the CTI as follows, in line with Squalli and Wilson’s (2011) suggestion:

4 $$CTI = \frac{{\left( {X + M} \right)_{i} }}{{\frac{1}{n}\mathop \sum \nolimits_{j = 1}^{n} \left( {X + M} \right)_{j} }} \frac{{\left( {X + M} \right)_{i} }}{{GDP_{i} }},$$

where *i* denotes South Africa; *j* reflects its trading partners; *X* represents exports; and *M* denotes imports. In Eq. (4), the first segment captures the world trade share, whereas the second portion accounts for South Africa’s trade share.

### Variables and data sources

We utilize yearly time-series data from 1960 to 2020. CO_2_ emissions, which is the dependent variable in this study, are used as a proxy for environmental quality. We employ the scale effect to capture income and the TE to represent the square of income to confirm the validity of the EKC hypothesis. A fiscal decentralization index based on expenditure and revenue decentralization, computed using principal component analysis, captures the ratio of spending/revenue shares to general government expenditure and revenue. Industrial value-added to GDP (IGDP), trade openness (OPEN) using a CTI as shown above, FDI, EC, and TECH utilizing the GDP are all control variables used in this empirical research.

The variables that were considered in the relationship between fiscal decentralization and environmental quality have the following justifications:

First, economic growth is used in the investigation because the EKC hypothesis uses an inverted U-shaped connection to predict a turning point between economic growth and environmental degradation. This result indicates that economic progress initially coincides with environmental deterioration. However, after crossing a certain threshold, economic progress is compatible with ecological sustainability (Zeraibi et al. 2022). That is, environmental issues will eventually be resolved owing to economic expansion, innovative technology, and a shift in the economy toward services and light manufacturing (Liu et al. 2022). The early-stage industrialization that necessitates extensive use of fossil fuels causes environmental destruction to grow initially. However, deterioration declines beyond a certain per capita income threshold (a turning point). As the economy shifts from being industrially oriented to being service sector focused, educational levels grow, and thus, environmental consciousness develops. Therefore, economic growth is a crucial instrument for analyzing a country’s development and progress; consequently, it has got much attention from scholars for decades (Naqvi et al. 2021; Sun et al. 2021). The reason is that faster economic growth necessitates more mineral wealth and energy utilization (particularly conventional sources of energy), which are critical to a country’s rate of development and sustainable changes (Minlah and Zhang 2021). However, such utilization has harmful ecological repercussions, such as anthropogenic climate change and the associated environmental degradation. Prior studies (Ahmad et al. 2021c; Liu, 2021; Isik et al. 2021) in energy and environmental economics elaborated that economic growth (as represented by the scale effect) is incorporated in this study to track the ecological effect of income.

Second, the square of economic growth (as expressed by TE) is included to assess the validity of the EKC hypothesis in the instance of South Africa.

Third, energy is employed just as frequently as capital and labor and is regarded as the most important input in manufacturing. As energy usage in the industry is so pervasive, uninterrupted energy sources are essential to maintaining and raising the current level of output and living standards. EC is considered a prerequisite for long-term economic development in the manufacturing process, although any deficit in energy output has a detrimental impact on economic growth. The inspiring society recognizes that energy use is a significant contribution to CO_2_ emissions, which are the principal cause of GHGs. Several works, such as Durrani et al. (2021), Ahmad et al. (2021a), and Li et al. (2021a, b, c), have considered energy usage to capture the influence of energy utilization on environmental degradation. As the energy industry accounts for 75% of worldwide GHG emissions (Udeagha and Breitenbach 2023a), EC is included to contribute to rising emissions levels.

Fourth, the following is a summary of the connection between FDI influx and environmental contamination in host nations. According to the “pollution haven hypothesis” (PHH), emerging nations are more likely to draw FDI by decreasing environmental standards in the early stages of economic growth to expand their economies quickly. However, using this strategy to draw capital results in shifting filthy industries—high-pollution, high-energy-consumption industries—from industrialized to emerging nations. This case raises the pollution level in emerging nations. The host nation (region) turns into a “pollution sanctuary” for industrialized nations (Musah et al. 2022). The contrarian position is that FDI defies the PHH. Local businesses benefit from the sophisticated industrial and environmental technology offered by FDI. Consequently, local businesses lower the degree of environmental degradation in their industry and the entire region through learning, competition, and demonstration effects (Chen et al. 2022). The scale, structural, and technical effects of FDI on the environment of the host nation may be separated into positive and negative effects (Chaudhry et al. 2022). FDI, while critical to a nation’s economic formation and development, particularly where local resources are insufficient to meet domestic financial commitments, has the potential to degrade the host nation’s quality of the environment (Shinwari et al. 2022). As a result, the research utilizes FDI to assess its ecological impact, as suggested by Anser et al. (2021a, b) and Rehman et al. (2021), because FDI promotes greater economic enterprises and thus could intensify emissions and lead to environmental degradation (Ahmad et al. 2021b).

Fifth, Copeland and Taylor (1994) and Grossman and Krueger (1995) established the theoretical foundation for the environmental impact of trade liberalization. Antweiler et al. (2001) highlighted the numerous elements impacting carbon emissions and how trade openness may have an impact on the environment and later expanded it. Thus, the composition, technique, and scale effects of environmental impacts of trade openness are separated out in the study. The degree of environmental deterioration is determined by the structural constitution of a nation’s industrial production. Therefore, the composition effect indicates this structural composition’s environmental impact. Environmental pollution will always be higher in a nation with a more carbon-intensive production structure than with a less carbon-intensive production structure. Therefore, the type of economy and its organizational structure influence the degree of environmental quality in that nation. From another aspect, the scale effect is an impact on emissions brought on by an increase in income. Owing to intense manufacturing, environmental quality declines as income rises. The technique impact results from the implementation of environmental regulations, which compel the private sector to use more modern, cleaner, and environmentally friendly industrial methods, thereby enhancing environmental quality. The TE leads to a greater quality of the environment owing to peoples’ inclination toward a clean environment and the implementation of stricter environmental rules as income rises. Therefore, trade openness is included to track the influence of trade openness on environmental quality in South Africa, as suggested by Li et al. (2021a, b, c), Khan and Ozturk (2021), Alvarado et al. (2021), and Ahmad et al. (2022).

Sixth, TECH is viewed as a critical component in reducing energy usage, improving energy efficiency, and enhancing the ecological environment (Ahmad and Wu 2022; Fareed et al. 2022). The use of technological advancement is necessary to enable sustainable industries and enhance the sustainability of the environment. Therefore, in line with Can et al. (2021), Ahmad and Wu (2022), and Fareed et al. (2022), the present study considers TECH among the key drivers of environmental quality to investigate its contribution, as this would help to modify forms of energy, including renewable sources from inefficient to more ecologically sound sources.

Finally, the ecological and overall total emissions of the nation are essential aspects of the nation’s economic vulnerability to industrialization (Rehman et al. 2021). Mineral wealth is diffused very quickly as industrialization progresses, which has an influence on the overall living standard of the burgeoning population and the ecosystem. Therefore, as indicated by Tian et al. (2014) and Hossain (2011), industrial value-added is regarded as one of the major factors in this study to analyze its influence on ecological quality.

Table 2 presents a summary of the definition of variables and the sources of data.

**Table 2:** Definition of variables and data sources

Variable	Description	Expected sign	Source
CO_2_	CO_2_ emissions (kg per 2015 US$ of GDP)	N/A	WDI
EC	Energy consumption, million tonnes oil equivalent	Positive	BP Statistical Review of World Energy
FISD	Fiscal decentralization is computed by an index from expenditure and revenue decentralization capturing the ratio of expenditure/revenue shares to the general government expenditure and revenue. This paper uses a principal component analysis to compute an index for fiscal decentralization	Positive or negative	IMF
TECH	Technological innovation measured by gross domestic spending on R&D (% GDP)	negative	WDI
OPEN	Trade openness computed as composite trade intensity introduced by Squalli and Wilson (2011) capturing trade effect	Positive or negative	WDI, Authors
SE	Real GDP per capita capturing scale effect	Positive	WDI
TE	Real GDP per capita squared capturing technique effect	Negative	WDI, Authors
FDI	Foreign direct investment, net inflows (% of GDP)	Positive	WDI
IGDP	Industry, value added (% of GDP)	Positive or negative	WD

*N/A* Not available; *WDI* World Development Indicator; *IMF* International Monetary Fund

### Narayan and Popp’s structural break unit root test

Before employing the novel dynamic ARDL simulations model, we first conduct a unit root test on the variables to investigate their order of integration. To do this, the study used the Kwiatkowski–Phillips–Schmidt–Shin (KPSS), Augmented Dickey–Fuller (ADF), Phillips–Perron (PP), and Dickey–Fuller GLS (DF-GLS) tests. The study employs the strategy recommended by Narayan and Popp (2010) to deal with two structural breaks in the dataset as they are common, and failing to address them could result in prejudiced and unreliable outcomes.

### ARDL bound testing approach

We use the bounds test to examine the long-term relationship between fiscal decentralization and environmental quality while adjusting for other variables. We provide the ARDL bound testing technique, following Pesaran et al. (2001):

5 $$\begin{aligned} \Delta In{\text{CO}}_{2t} & = \gamma_{0} + \pi_{1} In{\text{CO}}_{2t - i} + \pi_{2} InSE_{t - i} + \pi_{3} InTE_{t - i} + \pi_{4} InFISD_{t - i} \\ & \quad + \pi_{5} InTECH_{t - i} + \pi_{6} InEC_{t - i} + \pi_{7} InFDI_{t - i} + \pi_{8} InOPEN_{t - i} + \pi_{9} InIGDP_{t - i} \\ & \quad + \mathop \sum \limits_{i = 1}^{q} \gamma_{1i} \Delta In{\text{CO}}_{2t - i} + \mathop \sum \limits_{i = 1}^{q} \gamma_{2i} \Delta InSE_{t - i} + \mathop \sum \limits_{i = 1}^{q} \gamma_{3i} \Delta InTE_{t - i} + \mathop \sum \limits_{i = 1}^{q} \gamma_{4i} \Delta InFISD_{t - i} \\ & \quad + \mathop \sum \limits_{i = 1}^{q} \gamma_{5i} \Delta TECH_{t - i} + \mathop \sum \limits_{i = 1}^{q} \gamma_{6i} \Delta EC_{t - i} + \mathop \sum \limits_{i = 1}^{q} \gamma_{7i} \Delta InFDI_{t - i} + \mathop \sum \limits_{i = 1}^{q} \gamma_{8i} \Delta InOPEN_{t - i} \\ & \quad + \mathop \sum \limits_{i = 1}^{q} \gamma_{9i} \Delta InIGDP_{t - i} + \varepsilon_{t} ,{ } \\ \end{aligned}$$

where $\Delta$ signifies the first difference of InFISD, InIGDP, InOPEN, InFDI, InEC, InTECH, InTE, InSE, and InCO_2_, and $\varepsilon_{t}$ represents the white noise. The null hypothesis $(H_{0} :\pi_{1} = \pi_{2} = \pi_{3} = \pi_{4} = \pi_{5} = \pi_{6} = \pi_{7} = \pi_{8} = \pi_{9} = 0$) versus the alternative hypothesis $(H_{1} :\pi_{1} \ne \pi_{2} \ne \pi_{3} \ne \pi_{4} \ne \pi_{5} \ne \pi_{6} \ne \pi_{7} \ne \pi_{8} \ne \pi_{9} \ne 0)$ is tested.

The estimable ARDL equation for the long run is stated as follows:

6 $$In{CO2}_{t}={\beta }_{0}+\sum_{i=1}^{q}{\omega }_{1}{InCO2}_{t-i}+\sum_{i=1}^{q}{\omega }_{2}{InSE}_{t-i}+\sum_{i=1}^{q}{\omega }_{3}{InTE}_{t-i}+\sum_{i=1}^{q}{\omega }_{4}{InFISD}_{t-i}+\sum_{i=1}^{q}{\omega }_{5}{InTECH}_{t-i}+\sum_{i=1}^{q}{\omega }_{6}{InEC}_{t-i}+\sum_{i=1}^{q}{\omega }_{7}{InFDI}_{t-i}+\sum_{i=1}^{q}{\omega }_{8}{InOPEN}_{t-i}+\sum_{i=1}^{q}{\omega }_{9}{InIGDP}_{t-i}+{\varepsilon }_{t}.$$

In Eq. (6), $\omega$ represents the variance of the variables in the long run. The SBIC is used to identify the proper lags. Thus, the short-run error correction equation is:

7 $$\Delta In{\mathrm{CO}}_{2t}={\beta }_{0}+\sum_{i=1}^{q}{\pi }_{1}\Delta {In\mathrm{CO}}_{2t-i}+\sum_{i=1}^{q}{\pi }_{2}\Delta {InSE}_{t-i}+\sum_{i=1}^{q}{\pi }_{3}\Delta {InTE}_{t-1}+\sum_{i=1}^{q}{\pi }_{4}{\Delta InFISD}_{t-i}+\sum_{i=1}^{q}{\pi }_{5}{\Delta InTECH}_{t-i}+\sum_{i=1}^{q}{\pi }_{6}\Delta {InEC}_{t-1}+\sum_{i=1}^{q}{\pi }_{7}{In\Delta FDI}_{t-1}+\sum_{i=1}^{q}{\pi }_{8}\Delta {InOPEN}_{t-1}+\sum_{i=1}^{q}{\pi }_{9}\Delta {InIGDP}_{t-1}+{\varnothing EC{T}_{t-i}+\varepsilon }_{t}.$$

In Eq. (7), $\pi$ represents the variance of the variables in the short run, whereas the error correction component is denoted by error correction term (ECT), signifying the speed of disequilibrium adjustment. We also run various diagnostic tests to ensure the model is stable. Finally, the cumulative sum of squares of recursive residu als (CUSUMSQ) and the cumulative sum of recursive residuals (CUSUM) are used to assess structural stability.

### Dynamic ARDL simulations

On the one hand, past studies of the connection between fiscal decentralization and environmental quality have frequently used the standard ARDL framework proposed by Pesaran et al. (2001). This framework produces long- and short-run estimations. The ARDL technique is extensively used in energy, economic, and environmental research owing to its statistical benefits. The ARDL framework is particularly well suited, sturdy, and effective in the situation of a smaller sample (Pesaran et al. 2001). The ARDL technique does not have an autocorrelation problem, and the endogeneity concern is very well handled by selecting the appropriate lag duration (Langnel and Babington, 2020). Moreover, whether the variables under investigation are stationary at level I(0) or the first difference I(1), the ARDL technique may be applied. This method also offers the error correction model’s long- and short-run cointegration parameters in a single equation. On the other hand, the novel dynamic ARDL simulation model for time-series data has garnered considerable attention in environmental and energy economics. The framework effectively eliminates the difficulties associated with evaluating the estimates derived using the standard ARDL method for assessing long- and short-run coefficients of variables studied. Thus, the dynamic ARDL simulation methodology has gained prominence as a convincing technique to derive practical conclusions from time-series models with nonintuitive or “hidden” coefficients (Jordan and Philips 2018). Accounting for other factors, the novel dynamic ARDL simulations strategy effectively calculates, reproduces, and automatically visualizes the exact positive and negative shocks in independent and response variables. When employing the dynamic ARDL simulations methodology, the dependent variable should be stationary at the first difference. Second, the order of integration for the independent variable in the model cannot be larger than I(1). Even if the investigation does not require the difficult I(0)/I(1) judgment, all explanatory variables should be evaluated for explosiveness or seasonal stationarity of the variables (Jordan and Philips 2018). Therefore, implementing this unique technique in this study produces accurate and credible outcomes. As the parameter matrix has a multivariate normal distribution, the dynamic ARDL error correction algorithm in this study considers 1000 simulations. Furthermore, the graphs are used in this study to evaluate the actual changes in the explanatory variables, including their influence on the response variable. Moreover, this resourceful approach has been used in various experimental investigations to explore the short- and long-run association between the variables in question. For instance, Pata and Isik (2021) used this technique to look at the effects of energy intensity, per capita income, natural resource rent, and human capital on the load capacity factor in China from 1981 to 2017, focusing on environmental challenges on the demand and supply sides. Li et al. (2022) employed this framework to explore the relationship between income disparity and ecological sustainability by including the influence of human development and global economic integration in the framework. Similarly, Khan and Ozturk (2021) used the novel ARDL simulations framework to investigate the influence of technology in enhancing ecological integrity in the US and China. The following is a description of the novel dynamic ARDL simulations model:

8 $$\Delta In{CO2}_{t}={\alpha }_{0}+{\rho }_{0}{InCO2}_{t-1}+{\varphi }_{1}\Delta {SE}_{t}+{\rho }_{1}{SE}_{t-1}+{\varphi }_{2}\Delta {TE}_{t}+{\rho }_{2}{TE}_{t-1}+{\varphi }_{3}\Delta {FISD}_{t}+{\rho }_{3}{FISD}_{t-1}+{{\varphi }_{4}\Delta {TECH}_{t}+{\rho }_{4}{TECH}_{t-1}+\varphi }_{5}\Delta {EC}_{t}+{\rho }_{5}{EC}_{t-1}+{\varphi }_{6}\Delta {FDI}_{t}+{\rho }_{6}{FDI}_{t-1}+{\varphi }_{7}\Delta {OPEN}_{t}+{\rho }_{7}{OPEN}_{t-1}+{\varphi }_{8}\Delta {IGDP}_{t}+{\rho }_{8}{IGDP}_{t-1}+\delta EC{T}_{t-1}+{\varepsilon }_{t.}$$

### FDC test

The most effective and efficient testing paradigm given by Breitung and Candelon (2006), the current article contributes to the existing literature on the link between fiscal decentralization and environmental quality. As a result, the FDC framework allows for appropriate accounting of persistent causation between variables across long, short, and medium timeframes. In addition, the FDC approach is used as a robustness check in this research. To the best of our knowledge, previous research on the link between fiscal decentralization and environmental quality, notably in South Africa, has overlooked this comprehensive testing approach.

## Empirical results and their discussion

### Summary statistics

The descriptive analysis of the variables used in this study is analyzed before the findings are discussed by using the FDC technique. Table 3 presents a statistical summary, demonstrating that CO_2_ emissions have an arithmetic mean of 0.264. The TE has an average mean of 60.316, which is larger than the average mean of the other variables. With an average value of 13.203, FDI comes in second. In addition to summarizing the statistical information, Table 3 uses kurtosis to characterize the peaks and the Jarque–Bera diagnostic report to validate the normality of our time-series data. Table 3 shows an upward trend in TECH, industrial value-added, FDI, EC, trade openness, and scale effect but a negative trend in TE. The TE exhibits the most change of any variables, indicating that it is very volatile. CO_2_ emissions are much steadier than the TE, indicating that CO_2_ emissions are much less variable. Furthermore, there are far bigger variances in trade openness (OPEN), SE, and TECH. According to Jarque–Bera statistics, our data series are similarly regularly distributed.

**Table 3:** Descriptive statistics. *Source*: Authors’ calculations

Variables	Mean	Median	Maximum	Minimum	Std. Dev	Skewness	Kurtosis	J-B Stat	Probability
CO_2_^b^	0.264	0.238	0.477	0.084	0.120	0.217	1.652	4.682	0.196
SE^c^	7.706	7.959	8.984	6.073	0.843	-0.511	2.156	4.102	0.129
TE^d^	60.316	63.754	80.717	36.880	12.663	-0.387	2.082	3.422	0.181
FISD^e^	8.405	7.851	9.604	4.801	0.183	-0.418	1.401	3.154	0.101
TECH^f^	9.360	9.255	10.545	8.210	0.766	0.082	1.634	4.499	0.105
EC^g^	4.220	4.422	4.840	3.177	0.527	-0.558	1.921	5.621	0.160
FDI^h^	13.203	13.286	14.659	11.913	0.738	0.056	2.463	0.702	0.704
IGDP^i^	3.513	3.580	3.813	3.258	0.161	-0.215	1.697	4.474	0.107
OPEN^j^	6.060	6.512	7.665	2.745	1.329	0.636	2.077	5.757	0.156

^a^Data source: the data was collected from World Development Indicators (see Table 2 for more details)

^b^The unit of CO_2_ emissions was kg per 2015 US$ of GDP

^c^SE: scale effect denoting real GDP per capita, the unit of SE was current US$

^d^TE: technique effect capturing the square of real GDP per capita, the unit of TE was current US$

^e^FISD: Fiscal decentralization, the ratio of own revenues/expenditures to general government revenues/expenditures is used to generate an index using principal component analysis (PCA)

^f^TECH: technological innovation, the unit of TECH was in % of GDP

^g^EC: energy consumption, the unit of EC was kg of oil equivalent per capita

^h^FDI: foreign direct investment, the unit of FDI was in % of GDP

^i^IGDP: Industry, value added, the unit of IGDP was in % of GDP

^j^OPEN: trade openness, the unit of OPEN was in % of GDP

### Order of integration of the respective variables

Table 4 summarizes the findings of DF-GLS, PP, ADF, and KPSS, demonstrating that after initial differencing, all nonstationary variables become stationary at *I*(1). Thus, all of the series under consideration are *I*(1) or *I*(0), and none are *I*(2). The typical unit root tests mentioned above do not consider structural breaks. Therefore, this research employs a testing technique that can account for two structural breaks in the variables. In the right-hand panel of Table 4, the findings of Narayan and Popp’s unit root test with two structural breaks are also presented. The null hypothesis of the unit root cannot be rejected based on empirical results. Thus, the findings suggest that all data series are integrated of order one in the presence of structural breaks and a potential application for the dynamic ARDL bound testing approach.

**Table 4:** Unit root analysis. *Source*: Authors’ calculations

Variable	Dickey-Fuller GLS	Phillips-Perron	Augmented Dickey-Fuller	Kwiatkowski-Phillips-Schmidt-Shin	Narayan and Pop (2010) Unit Root Test			
	(DF-GLS)	(PP)	(ADF)	(KPSS)	Model 1		Model 2	
Level	Test—Statistics value				Break-Year	ADF-stat	Break-Year	ADF-stat
InCO_2_	− 0.570	− 0.464	− 1.152	0.966	1982:1985	− 3.132	1987:1994	− 8.160***
InSE	− 0.116**	− 0.079	− 1.308	0.833***	1979:1988	− 2.914	1982:1990	− 7.601***
InTE	− 0.112*	− 0.076	− 1.268	0.848***	1979:1990	− 1.939	1982:1994	− 6.791***
InFISD	− 0.140**	− 0.067	− 1.127	0.692***	1980:1991	− 2.510	2007:2013	− 7.153***
InTECH	− 0.254***	− 0.284***	− 2.999	0.255***	1995:2000	− 4.318	2008:2011	− 7.821***
InEC	− 0.011	− 0.014	− 0.366	1.300***	1982:1989	− 4.372**	1985:1991	− 8.521***
InFDI	− 0.032*	− 0.001	− 0.012	0.640	2001:2006	− 2.021	2004:2010	− 8.362***
InOPEN	− 0.072	− 0.082	− 1.335	1.080*	1996:2001	− 3.053	2003:2009	− 7.318***
InIGDP	− 0.046	− 0.071*	− 1.718	1.060**	1972:1985	− 3.815	1982:1991	− 7.521***

First difference					Critical value (1%, 5%, and 10%)			
$\Delta$InCO_2_	− 0.995***	− 0.996***	− 7.176***	0.705***	1999:2005	− 4.801**	1980:1991	− 5.832***
$\Delta$InSE	− 0.695***	− 0.707***	− 5.319***	0.585***	1983:1997	− 5.831***	1985:1995	− 6.831***
$\Delta$InTE	− 0.694***	− 0.707***	− 5.316***	0.589***	1991:2000	− 8.531***	1987:1996	− 5.893***
$\Delta$InFISD	− 0.183***	− 0.485***	− 7.814***	0.602***	1983:1999	− 5.714***	1982:2006	− 5.517***
$\Delta$InTECH	− 1.023***	− 1.034***	− 7.473***	0.424***	1999:2003	− 4.841**	2006:2010	− 5.983***
$\Delta$InEC	− 1.105***	− 1.121***	− 8.142***	0.586***	1985:1993	− 5.921***	1989:1997	− 7.942***
$\Delta$InFDI	− 0.207**	− 0.209**	− 6.443***	0.609***	2005:2008	− 6.831***	2001:2008	− 6.973***
$\Delta$InOPEN	− 0.935***	− 0.938***	− 6.699***	0.626***	1996:2004	− 6.842**	2001:2007	− 8.942***
$\Delta$InIGDP	− 0.799***	− 0.801***	− 5.878***	0.431***	1975:1990	− 7.742***	1988:1992	− 7.892***

*, ** and *** denote statistical significance at 10%, 5% and 1% levels, respectively. MacKinnon’s (1996) one-sided p-values. Lag Length based on SIC and AIC. Probability-based on Kwiatkowski–Phillips–Schmidt–Shin (1992). The critical values for Narayan-Popp unit root test with two breaks are followed by Narayan and Pop (2010). All the variables are trended

### Lag length selection results

Table 5 summarizes the results of several lags selection test criteria. The usage of HQIC, AIC, and SIC as the most common methods for determining acceptable lags has been reported in empirical research. For lag selection, SIC is employed in this study. Lag one, according to this method, is appropriate for our model. The reason is that, unlike other methods, when SIC is applied, the lowest result is achieved at lag one.

**Table 5:** Lag length criteria. *Source*: Authors’ calculations

Lag	LogL	LR	FPE	AIC	SC	HQ
0	161.450	NA	3.1e−12	− 7.594	− 7.331	− 6.493
1	614.091	801.28	1.3e−18	− 31.195	− 20.094*	− 20.390*
2	645.095	121	1.2e−18	− 31.388	− 18.448	− 19.877
3	742.750	121.32	1.1e−18*	− 31.759	− 16.981	− 19.544
4	761.113	103.72*	1.4e−18	− 32.350*	− 15.733	− 19.430

^*^ indicates lag order selected by the criterion

### Cointegration test results

Table 6 displays the outcomes of the cointegration test utilizing surface-response regression developed by Kripfganz and Schneider (2018). The F- and t-statistics are larger than the upper bound critical values at different significance levels, so we reject the null hypothesis. The experimental data show that the factors under consideration are therefore cointegrated. Given that the traditional test used above does not consider the structural breaks in the data, the Gregory–Hansen test of cointegration with regime changes is also utilized to test for cointegration in the presence of endogenous structural breaks. The findings, which are presented in Table 10 in the appendix, demonstrate that the considered variables are cointegrated at the breaking point. Our results are in line with the previously identified cointegrating connection, which made the supposition that the data are free of structural breaks.

**Table 6:** ARDL bounds test analysis

Test statistics	Value	K	${H}_{0}$	${H}_{1}$
F-statistics	13.337	8	No level relationship	Relationship exists
t-statistics	− 10.121			
Kripfganz &Schneider (2018) critical values and approximate p-values y				

Significance (%)	F-statistics		t-statistics		p-value F	
	1(0)	1(1)	1(0)	1(1)	1(0)	1(1)
10	2.12	3.23	− 2.57	− 4.04	0.000***	0.000***
5	2.45	3.61	− 2.86	− 4.38	p-value t	
1	3.15	4.43	− 3.43	− 4.99	0.000***	0.002**

*, ** and *** respectively represent statistical significance at 10%, 5% and 1% levels. The respective significance levels suggest the rejection of the null hypothesis of no cointegration. The optimal lag length on each variable is chosen by the Schwarz’s Bayesian information criterion (SBIC)

### Diagnostic statistics tests

The study applies several diagnostic statistical tests to confirm that our selected model is dependable and consistent. Table 7 presents their empirical findings. The empirical findings indicate that the chosen model is well-fitting as it passed all diagnostic tests. The Breusch Godfrey LM test confirms that the model is free of serial correlation and autocorrelation concerns. The Ramsey RESET test is employed, and data indicate that the model is correctly specified. The Breusch–Pagan–Godfrey and ARCH tests are used to determine if the model has signs of heteroscedasticity. According to the empirical data, heteroscedasticity is mild and not an issue. Finally, the Jarque–Bera test confirms that the model residuals are normally distributed.

**Table 7:** Diagnostic statistics tests

Diagnostic statistics tests	${X}^{2}$(p values)	Results
Breusch Godfrey LM test	0.3837	No problem of serial correlations
Breusch-Pagan-Godfrey test	0.2663	No problem of heteroscedasticity
ARCH test	0.6841	No problem of heteroscedasticity
Ramsey RESET test	0.5102	Model is specified correctly
Jarque–Bera Test	0.2732	Estimated residual are normal

*Source* Authors’ calculations

### Dynamic ARDL simulation model results

Table 8 shows the results of the dynamic ARDL simulation framework. Our results show that the scale effect (InSE) and the technique effect (InTE) have a positive and negative influence on environmental quality, respectively. The technique effect benefits the atmosphere, whereas the scale effect, which is a representation of economic expansion, damages ecological integrity. Therefore, the empirical conclusion confirms that the EKC theory is valid for South Africa. The outcomes are related to the fundamental change and technological advancement of the nation. Environmental regulations are implemented when public concern for the environment grows to promote the use of energy-efficient technologies and lessen emissions. These results support the observations of Udeagha and Breitenbach (2021), which demonstrate the validity of the EKC theory for the Southern African Development Community (SADC) from 1960 to 2014. Alharthi et al. (2021) had similar findings in the Middle East and North Africa, supporting the EKC theory in these countries. Our results concur with those of Ahmed et al. (2022), who remarked that Pakistan has an EKC since the income coefficient is positive. However, its quadratic component is negative from 1984 to 2017. Udeagha and Ngepah’s (2019) investigation of South Africa further confirmed our result. Similarly, based on balanced yearly panel data, Ahmad et al. (2021c) demonstrated that EKC existed in 11 developing economies from 1992 to 2014. Isik et al. (2020) validated this evidence in their analysis of G7 nations from 1995 to 2015 for France, but EKC was not valid in the US, the UK, Japan, Italy, Germany, and Canada. Additionally, according to Udeagha and Muchapondwa (2022b), EKC was found in South Africa from 1960 to 2020. Our evidence further supports those of Murshed (2021) for six South Asian economies. However, the results go against those of Minlah and Zhang (2021), who discovered that the EKC hypothesis was not valid for Ghana. The EKC hypothesis is invalid, as shown by similar findings of Ozturk (2015), Sohag et al. (2019), Tedino (2017), and Mensah et al. (2018).

**Table 8:** Dynamic ARDL simulations analysis

Variables	Coefficient	St. Error	t-value
Cons	− 1.1813	1.2816	− 0.93
InSE	0.2301***	0.1817	4.57
$\Delta$ InSE	0.3905***	0.2718	2.75
InTE	− 0.6021**	0.8245	− 2.37
$\Delta$ InTE	− 0.7363	0.1417	− 1.78
InFISD	− 0.3020***	0.1202	3.51
$\Delta$ InFISD	− 0.2031***	0.0671	2.53
InTECH	− 0.7223***	0.5871	− 3.24
$\Delta$ InTECH	− 0.2358**	0.0698	− 2.62
InEC	0.2741***	0.1762	3.98
$\Delta$ InEC	0.5972*	0.1719	1.98
InFDI	0.9015	0.0810	1.12
$\Delta$ InFDI	0.2881**	0.2657	2.59
InOPEN	0.1805***	0.0487	5.39
$\Delta$ InOPEN	− 0.3082**	0.0570	− 2.53
InIGDP	0.3434**	0.1577	2.17
$\Delta$ InIGDP	0.5373	0.2309	0.23
ECT(− 1)	− 0.8392***	0.1286	− 3.05
R-squared	0.7861		
Adj R-squared	0.7705		
N	55		
p val of F-sta	0.0000***		
Simulations	1000		

*, ** and *** denote statistical significance at 10%, 5% and 1% levels, respectively

*Source* Authors’ calculations

The estimated fiscal decentralization (InFISD) long- and short-run coefficients are statistically significant and negative. In the short and long run, fiscal decentralization is sustainable and environmentally friendly. Fiscal decentralization results in a 0.302% and 0.203% reduction in CO_2_ emissions in the long and short run, respectively. As a result, we deduce that the green paradox exists in the case of South Africa, which might be explained by the fact that, owing to strong local environmental legislation and enhanced state permission, South Africa’s environmental quality increases through the decentralization process. Hence, fiscal decentralization is important for South Africa to meet its low CO_2_ emissions objectives. Several pieces of evidence of the “race to the top” hypothesis in South Africa exist, where the government improves its environment by adopting a “beggar-thy-neighbor” policy to move polluting businesses to neighboring countries and thereby reap the advantages of its decentralization programs. However, a delineation of duties at various levels of government is required to realize the energy-saving functions of fiscal spending. Fiscal decentralization encourages new industrial capabilities while improving environmentally friendly technologies in the economic system. Local authorities’ preferences mirrored local demands, with a preference for public goods and a sustainable environment. The administrative authority, quality of institutions, environmentally biased technologies, green energy sources, liberty, and finances may all impact ecological quality owing to fiscal decentralization. According to the doctrine of fiscal decentralization, municipalities have a better grasp of their residents’ basic needs than the central government, as pointed out by Millimet (2003). Moreover, fiscal decentralization has a beneficial impact on ecological sustainability. Fiscal decentralization has a substantial influence on environmental quality, considering that local councils have greater access to resources and authority to safeguard the environment with greater amounts of decentralization. The results further illustrate that municipalities have a deeper understanding of the environmental condition and invest so much in it. Fiscal decentralization enables the federal authorities to delegate greater decision-making authority to provinces and municipalities, which has a positive impact on ecological protection. Decentralization enhances political rivalry among subnational governments over time; municipal authorities exhibit protectionist impulses, thereby raising environmental standards. These findings corroborate those of Xu (2021), who examined the relationship between fiscal decentralization and environmental management efficiency in China. They observed that fiscal decentralization significantly promotes environmental governance and efficiency, thereby reducing the rate of environmental deterioration in the country. The assistance of local governments for TECH initiatives is primarily responsible for this good driving impact. Expenditure and revenue decentralization would allow local authorities to gain information advantages and other features to increase local governance efficacy. Tufail et al. (2021), who demonstrated that fiscal decentralization enhances the quality of the environment by cutting CO_2_ emissions in seven highly fiscally decentralized OECD nations, back up this empirical finding. City authorities use energy-saving and pollution-mitigation programs in highly decentralized areas to guarantee that their energy-saving and carbon-neutrality objectives are accomplished. This empirical evidence is also consistent with the findings of Rani (2021) that fiscal decentralization aids Asian countries in reducing pollution. The author argued that fiscal decentralization might be increased through human capital and green innovation to attain ecological sustainability in the Asian block examined. Jain et al. (2021) drew a similar conclusion for nine Asian countries. Fiscal decentralization boosts “ecofriendly economic growth” via city authorities, which benefits the environment indirectly. This event encourages the manufacturing innovation process while strengthening environmentally friendly technologies in the industry. Our finding is also aligned with Du and Sun (2021), who observed that fiscal decentralization improves environmental quality through environmental-biased technological improvements. This finding is further supported by Su et al. (2021), who observed that fiscal decentralization could help accelerate trading and investment enterprises by improving ecological integrity. However, the findings contradict those of Xiao-Sheng et al. (2021), who showed that fiscal decentralization is harmful to the environment in Chinese cities. Similarly, Xia et al. (2021) revealed that fiscal decentralization significantly increases CO_2_ emissions inside and beyond the area. Furthermore, Lin and Zhou (2021) discovered that fiscal decentralization had a detrimental impact on environmental performance in China’s economically developed and eastern areas. Equally, Iqbal et al. (2021) looked into the roles of fiscal decentralization, environmental innovation, and export diversification in realizing the carbon reduction objective for 37 OECD countries. They found that fiscal decentralization and export diversification degrade the quality of the environment, whereas environmental innovation helps to improve the sustainability of the environment.

Additionally, technological innovation (InTECH) has a beneficial long- and short-term impact on CO_2_, suggesting that South Africa’s decreased CO_2_ levels can be attributed to a rise in TECH. This outcome supports Villanthenkodath and Mahalik (2022), who revealed that economic growth brought forth by technological advancement led to decreased pollution levels. The endogenous growth hypothesis, which contends that technological development boosts a nation’s capacity to replace polluting resources with more environmentally friendly ones, lends credence to this case. According to Shahzad et al. (2022), technological innovation provides a pathway that enables a decrease in EC, an improvement in energy efficiency, and a considerable reduction in carbon emissions. Our findings concur with those of Ibrahim and Vo (2021), who revealed that innovative technologies improve ecological quality in Big Emerging Market (BEM) countries. Destek and Manga (2021) also noted that, between 1995 and 2016, BEM nations’ carbon emissions decreased owing to technological advancement. This evidence agrees with the assertation of Ahmad and Wu (2022) that ecoinnovation improved carbon emissions abatement in OECD economies from 1990 to 2017. An et al. (2021) also reached similar findings for the Belt and Road Host nations. Additionally, Guo et al. (2021) and Baloch et al. (2021) found similar results, demonstrating that technological improvement reduces CO_2_ in Asian and OECD nations, respectively. Given the significance of technical advancement, we can assume that a country’s CO_2_ emissions will be reduced with an improved industrial structure. Ahmad and Raza (2020) noted that green technologies are crucial for greener manufacturing in America, which is advantageous for modernizing the industrial structure. In addition, Altinoz et al. (2021) stressed the importance of innovation and the need for energy advances in raising environmental quality. Our results agree with those of Yang et al. (2021a, b) for Brazil, Russia, India, China, and South Africa’s (BRICS) economies and Anser et al. (2021a, b) for EU nations. The results, however, differ from those of Usman and Hammar (2021), who discovered that technological advancement increased carbon emissions in the Asia Pacific Economic Cooperation from 1990 to 2017. Furthermore, Can et al. (2021), who used economic complexity as a gauge of technological progress, concluded that TECH played a significant role in environmental degradation and increased EC in 10 newly industrialized countries between 1970 and 2014. In addition, Fareed et al. (2022) found that, in 27 European countries, from 1995 to 2018, innovation increased environmental deterioration while mitigating the negative effects of financial inclusion on environmental quality. Khattak et al. (2020) made similar findings for BRICS’s economies, Arshad et al. (2020) for the South and Southeast Asian area, Demir et al. (2020) for Turkey, Villanthenkodath and Mahalik (2022) for India, and Faisal et al. (2020) for BEM nations.

In South Africa, ecological deterioration is observed to be exacerbated by energy consumption (InEC). According to our empirical findings, South Africa’s ecological sustainability is severely affected by EC. Given that South Africa is a developing country that has historically relied on fossil fuels, this outcome is to be anticipated. The country relies on the energy sector, where coal usage accounts for the majority of production activities. South Africa’s coal reserves provide over 77% of the primary energy and 93% of the power output (Udeagha and Breitenbach 2023b). CO_2_ emissions in South Africa have grown dramatically over the years because of the constant rise in EC, which has serious environmental consequences and is a key contributor to global climate change. Therefore, an increase in energy use per capita is expected to result in higher levels of energy-related CO_2_ emissions. Thus, South Africa may one day be subject to severe environmental degradation if the problem of its dependence on fossil fuels is not resolved. The switch from using fossil fuels to comparatively renewables, such as geothermal energy, tidal power, bioenergy, wave energy, hydroelectric power energy, solar energy, biomass, nuclear energy, and nuclear energy, requires TECH. Thus, policymakers should work toward accomplishing this goal. These results concur with those of Shakib et al. (2022), who regarded the relationship among energy, economy, and environment and found that energy use accelerated environmental degradation in 42 Belt and Road Initiative developing nations. Similarly, Li et al. (2021a, b, c), who investigated the environmental effects of green investment and other key macroeconomic aggregates, found that energy investments deteriorate environmental quality. Equally, Liu et al. (2015a, b), who studied the environmental consequences of conventional and alternative EC in China using a system dynamics model from 2013 to 2020, found that the overall energy use and CO_2_ emissions have significantly increased. This observation is in line with Durrani et al. (2021), who utilized a multivariate causality approach to demonstrate how energy usage drove economic production processes and influenced environmental quality in Pakistan from 1972 to 2015. Furthermore, using 31 Chinese regional datasets, Ahmad et al. (2021a) found that EC was a significant driver of environmental degradation in China from 2005 to 2018. According to Hu et al. (2021), energy use raised carbon emissions in Guangdong, China. However, our empirical evidence conflicts with those of Baye et al. (2021), Irfan (2021), Hao et al. (2021), He et al. (2021), I. Khan et al. (2021b), and Ponce and Khan (2021), who observed that increasing energy usage was beneficial to the environment.

Additionally, foreign direct investment (InFDI) had a significantly positive effect on South Africa’s ecosystem. Other factors, such as access to cheap labor and relative vicinity to foreign investors, increase the attraction of FDI and the likelihood that this outcome will occur. The above reinforces the notion that South Africa continues to thrive and build its economy while considering the state of its environment. This notion backed up the argument made by certain opponents of FDI, particularly those worried about the long-term sustainability of developing countries. This finding strengthens the case for the PHH. Our findings are consistent with those of Copeland and Taylor (2013), who found that factories that produce dirty goods have moved to developing economies. They bring with them the industrialized economies’ environmental problems, thereby worsening the environmental degradation already present in these underdeveloped nations. As South Africa focuses on producing dirty goods, which significantly worsens the rising levels of environmental degradation, corrupt institutions and lax environmental norms have made the country dirtier. FDI inflows have undoubtedly contributed to South Africa’s metamorphosis into a highly polluting global factory that sells and markets a large portion of what it produces back into foreign markets. Our research demonstrates the characteristics of South Africa’s economy, which is among the continent’s fastest growing. This result supports the findings of Xue et al. (2021), which show that FDI inflows raise carbon emissions throughout the selected South Asian countries. Our results concur with those of Murshed et al. (2021a, b), who noted that, between 1990 and 2016, the increased air pollution in South Asian nations was a major factor in FDI’s significant role in accelerating environmental deterioration. Furthermore, our outcome is similar to that of Ahmad et al. (2021b), who used the Chinese aggregate dataset to show that FDI was a key factor contributing to ecological damage in China. Similarly, Rehman et al. (2021) corroborated our finding, demonstrating that positive and negative changes in FDI resulted in environmental deterioration in Pakistan from 1975 to 2017. This empirical finding is further supported by Udeagha and Ngepah (2022d, 2020). Our finding, however, contradicted those of Anser et al. (2021a, b), who found that FDI helped to decrease carbon impacts across nations from 1995 to 2018. In addition, Joshua et al. (2020) and Omri et al. (2014) observed that FDI contributed to improving ecological quality in South Africa and 54 countries investigated, respectively. This evidence is also consistent with Shinwari et al. (2022), who studied the ecological impacts of Chinese FDI on the Belt and Road Economies from 2000 to 2019. They concluded that Chinese FDI has made a significant contribution to improving the ecological quality of the aforementioned economies, whereas FDI from other countries is a major source of environmental degradation.

The estimated coefficient on trade openness (InOPEN) for the long run is statistically significant and positive, implying that a 1% rise in trade openness deteriorates environmental quality by 0.18%. This finding is similar to the findings of Baek et al. (2009), who claimed that trade openness considerably worsens environmental conditions in poor nations. Our empirical evidence suggests that South Africa’s openness to international goods markets is not environmentally friendly, particularly in the long run. This finding contrasts with the short-run results, which show that trade openness improves the nation’s environmental quality. Meanwhile, the long-term negative impact of openness on South Africa’s environmental quality unquestionably reinforces the country’s opposition to greater economic liberalization. The sort of exportable products that constitute the nation’s basket of international markets justifies this outcome. Continuous harvesting of these items to fulfill the rising worldwide markets has a substantial negative impact on South Africa’s ecological quality. Furthermore, our findings may be explained by the theoretical framework of Lopez (1994), which states that energy-intensive industries consuming a large amount of energy, mostly owing to trade liberalization, such as transportation and manufacturing, pollute the environment. Furthermore, our findings are consistent with Taylor’s (2004) PHH, which states that poor nations, such as South Africa, have a competitive edge in producing dirty products, whereas industrialized economies have a competitive edge in creating green commodities. Thus, affluent nations tend to export pollution to underdeveloped ones through international trade (Cole 2004; Wagner 2010). Our results are consistent with those of Chishti et al. (2022), who noted that trade openness significantly accelerated environmental deterioration in the Pakistani climatic condition by increasing air pollution. Khan et al. (2022a, b) obtained similar findings. They observed that trade openness helped considerably to accelerate environmental deterioration by increasing air pollution for the Next Eleven nations’ net fuel importers subpanel. Therefore, these results are consistent with the hypothesis that the Next Eleven countries have developed specializations in the production of goods and services that contribute to pollution owing to their historical reliance on dirty energy sources. Hence, the Next Eleven countries have significantly harmed the environment by expanding cross-border trade. As these countries’ trade openness indices are considerably greater than those of the net fuel-exporting Next Eleven countries, the negative environmental effects of international trade involvement are significantly more severe for the net fuel-importing Next Eleven countries. The results of our study concur with those of Murshed et al. (2020), who found that increased air pollution in South Asian nations was a major factor in the acceleration of environmental deterioration brought on by ICT trade openness. Similarly, Xin et al. (2022) found that trade openness worsens environmental quality in the USA. Our finding is also in agreement with the findings of Ahmad et al. (2019), who observed that international trade contributed to the worsening of environmental conditions in 29 Chinese provinces and cities from 1997 to 2016. Equally, Li et al. (2021a, b, c) backed our finding, indicating that trade openness exacerbated CO_2_ emissions, prolonging China’s attainment of ecological sustainability from 1989 to 2019. The authors further revealed that the structural break dummy combined with trade openness indicated that, in the post-2001 era, trade openness significantly worsened the sustainability of the environment in the wake of economic reform achieved after signing to be a member of the World Trade Organization. Our empirical result is also corroborated and consistent with the findings of Khan and Ozturk (2021), who found that developing nations tend to emit a high number of pollutants owing to their reliance on polluting sectors driven by international trade. Our findings are consistent with those of Khan et al. (2021a), who contend that trade openness is harmful and has significantly exacerbated Pakistan’s environmental situation. Ngepah and Udeagha (2019) observed similar results for the African area, as did Ngepah and Udeagha (2018) for Sub-Saharan Africa. However, this conclusion contradicted the findings of Alvarado et al. (2021), who showed that trade greatly decreased the ecological footprint of lower-middle-income nations from 1980 to 2016. A rationale for this outcome is that trade intensifies environmental pressure caused by human activity, particularly in environments with liberal environmental regulations. Our result further contradicted those of Ibrahim and Ajide (2021b), Ibrahim and Ajide (2022), and Ding et al. (2021), who reported that more trade openness improved environmental quality in G-20, 48 Sub-Saharan African, and G-7 nations, respectively. Udeagha and Breitenbach (2021) observed similar results for SADC nations. Similarly, Ahmad et al. (2022) found that an increase in the balance of trade improved environmental quality in Pakistan from 1970 to 2018.

For the industrial value-added share of GDP (InIGDP), the estimated coefficient is statistically significant and positive in the long run. This result indicates that the industrial sector growth contributes considerably to the long-run deterioration of South Africa’s environmental quality. South Africa’s expanding industrial sector is mostly to blame for the country’s rising CO_2_ emissions. South Africa has implemented several policies over the years to pursue structural transformation and industrialization to decrease poverty and promote equitable growth. The structural change of the economy from low-productivity agriculture to high-productivity industrialization is regarded as necessary for achieving long-term economic development, job creation, and poverty alleviation. However, South Africa’s expanding industrial sector has resulted in an increase in CO_2_ emissions. Large-scale industrialization and accompanying environmental change, including the influence on biodiversity, represent a danger to human survival through ecological functions, recreation, and basic requirements. Evidently, environmental degradation from many channels, notably industries, has a detrimental influence on the ecosystem that is permanent in nature and leads to the loss of natural habitats and valuable genetic resources. Our findings are congruent with those of Al Mamun et al. (2014) and Sohag et al. (2017), who found that growing industrial sectors were predominantly responsible for rising CO_2_ emissions in high-income non-OECD and upper middle-income countries and middle-income countries, respectively. Considering that many emerging economies are in transition, a harmony between rapid industrialization and environmental characteristics should be maintained to minimize the carbon emissions burden. According to Jahanger et al. (2022), industrialization increased CO_2_ emissions for the countries of Latin America and the Caribbean between 1990 and 2016. Tian et al. (2014) also revealed that industrialization is a significant contributor to CO_2_ emissions in the United States. The empirical evidence is further consistent with the findings of Li and Xia (2013), Shahbaz and Lean (2012), Garcia and Sperling (2010), Lin et al. (2009), Al-Mulali and Ozturk (2015), Asane-Otoo (2015), Nejat et al. (2015), Poumanyvong and Kaneko (2010), Hossain (2011), and Cherniwchan (2012). However, our result disagreed with Lin et al. (2015), who noted that there was no indication that the industrial sector expansion contributed to environmental damage in Nigeria. Similar works, such as Ewing and Rong (2008), Shahbaz et al. (2015), Wang et al. (2014), Lin et al. (2017), Kavzoǧlu (2008), Ahuti (2015), Dhami et al. (2013), Zhang and Lin (2012), Xu and Lin (2015), Zhou et al. (2013), Shafiei and Salim (2014), and Shahbaz et al. (2014a, b), revealed that industrial sector expansion was beneficial to the environment.

The model converges at a rate of almost 84% each year, as shown by the ECT, which is significant and negative. In the dynamic ARDL model, impulse response curves are employed to forecast how a regressed variable will change over time in reaction to an explanatory variable. The 75%, 90%, and 95% confidence intervals are indicated by the deep blue to light blue lines, whereas the dots represent the predicted value.

**Fig. 1:**
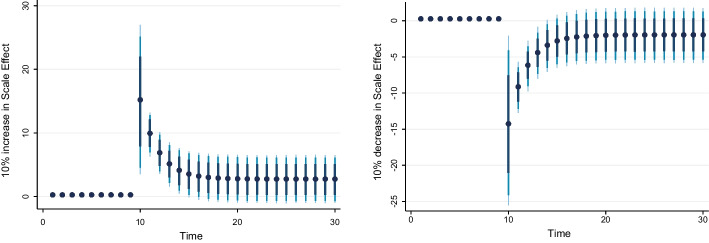
The Impulse Response Plot for Scale Effect (Economic Growth) and CO_2_ Emissions. Figure 1 presents an increase and a decrease by 10% in scale effect and its effect on CO_2_ emissions where dots denote average prediction value. The dark blue to light blue line shows 75, 90, and 95% confidence interval, respectively

Figure 1 shows the relationship between the scale effect and CO_2_ emission. In the short term, CO_2_ emissions rise steadily as the scale effect’s contribution increases by 10%. Additionally, each 10% reduction in the scale effect contribution results in a corresponding reaction in CO_2_ emissions. On the one hand, a persistent rise in the contribution of the scale effect causes CO_2_ emission to increase with time. On the other hand, any reduction in the scale effect’s contribution appears to mitigate its long-term negative effects on the ecosystem.

**Fig. 2:**
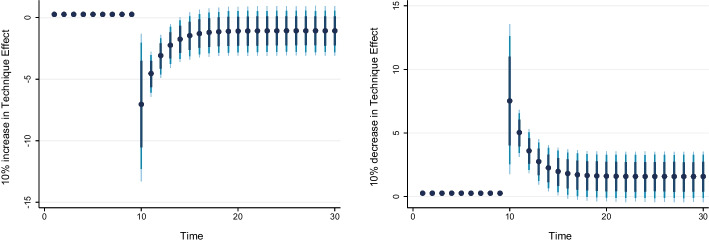
The Impulse Response Plot for Technique Effect and CO_2_ Emissions. Figure 2 presents an increase and a decrease by 10% in technique effect and its effect on CO_2_ emissions where dots denote average prediction value. The dark blue to light blue line shows 75, 90, and 95% confidence interval, respectively

Figure 2 depicts the impulse response curves for a 10% rise and reduction in technique effect. A 10% rise in technique effect lowers CO_2_ emissions, whereas a 10% drop results in environmental harm. With a drop in technique effect, CO_2_ emission grows over time; nevertheless, each 10% boost in technique effect results in a flat drop in CO_2_ emissions. However, as CO_2_ emission is still increasing, this improvement in TE cannot help the environment.

**Fig. 3:**
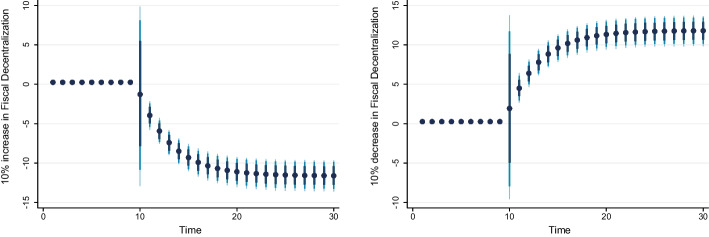
The Impulse Response Plot for Fiscal Decentralization and CO_2_ Emissions. Figure 3 presents an increase and a decrease by 10% in fiscal decentralization and its effect on CO_2_ emissions where dots denote average prediction value. The dark blue to light blue line shows 75, 90, and 95% confidence interval, respectively

Figure 3 shows the impulse response curves for a 10% rise and reduction in fiscal decentralization. Fiscal decentralization may be increased by 10% to reduce CO_2_ emissions, but it can also be decreased by 10% without having a favorable environmental impact. As fiscal decentralization decreases over time, CO_2_ emissions rise; nevertheless, with every 10% increase in fiscal decentralization, CO_2_ emissions go down by the same amount. However, this progress in fiscal decentralization might benefit the environment because CO_2_ emissions are still increasing.

**Fig. 4:**
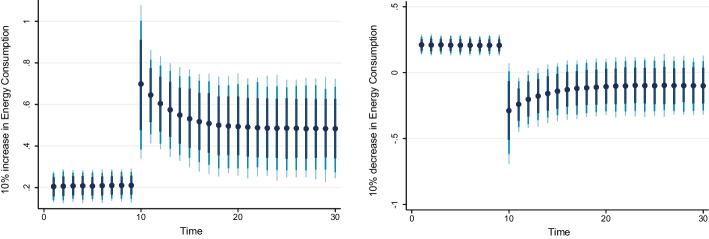
The Impulse Response Plot for Energy Consumption and CO_2_ Emissions. Figure 4 presents an increase and a decrease by 10% in energy consumption and its effect on CO_2_ emissions where dots denote average prediction value. The dark blue to light blue line shows 75, 90, and 95% confidence interval, respectively

Figure 4 illustrates the correlation between energy use and CO_2_ emission. Any boost in energy usage has a detrimental effect on the environment in the short term. However, any additional increases in energy consumption result in an aggravation of atmospheric quality over time, whereas a decrease in energy use helps to slow down environmental deterioration over time. The harm done to the environment, however, cannot be reversed.

**Fig. 5:**
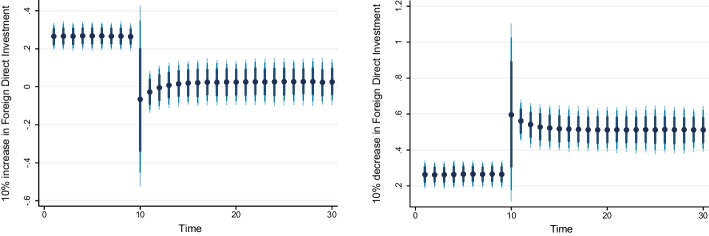
The Impulse Response Plot for Foreign Direct Investment and CO_2_ Emissions. Figure 5 presents an increase and a decrease by 10% in foreign direct investment and its effect on CO_2_ emissions where dots denote average prediction value. The dark blue to light blue line shows 75, 90, and 95% confidence interval, respectively

Figure 5 shows that FDI is predicted to have a negligible long-term influence on the environment. Furthermore, every 10% increase in FDI over the long term worsens ecological integrity. From another aspect, every reduction in FDI causes a reduction in CO_2_ emissions.

**Fig. 6:**
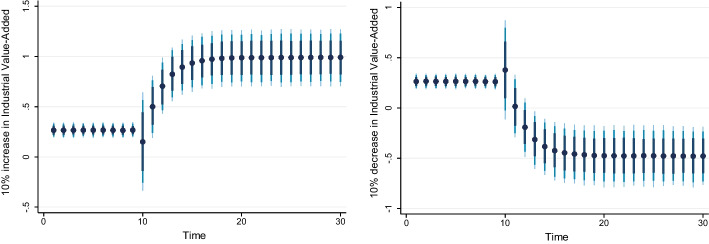
The Impulse Response Plot for Industrial Value-Added and CO_2_ Emissions. Figure 6 presents an increase and a decrease by 10% in industrial value-added and its effect on CO_2_ emissions where dots denote average prediction value. The dark blue to light blue line shows 75, 90, and 95% confidence interval, respectively

Figure 6 shows the expected trajectory of CO_2_ emissions in response to changes in CO_2_ emissions corresponding to industrial value-added. A 10% rise in industrial value-added has deleterious short- and long-term ecological effects. The beneficial shift over the long term, nevertheless, is more pronounced.

**Fig. 7:**
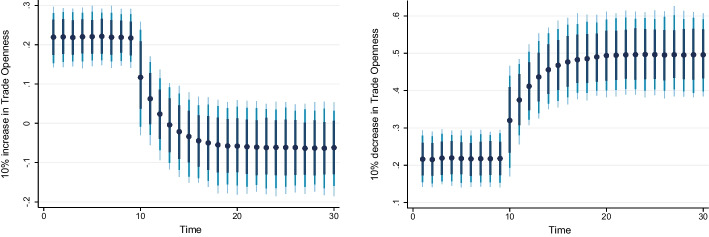
The Impulse Response Plot for Trade Openness and CO_2_ Emissions. Figure 7 presents an increase and a decrease by 10% in trade openness and its effect on CO_2_ emissions where dots denote average prediction value. The dark blue to light blue line shows 75, 90, and 95% confidence interval, respectively

A consistent rise in trade openness over the short run significantly reduces CO_2_ emissions, as shown in Fig. 7. By contrast, the long-term improvement in environmental quality is influenced by the fall in trade openness.

**Fig. 8:**
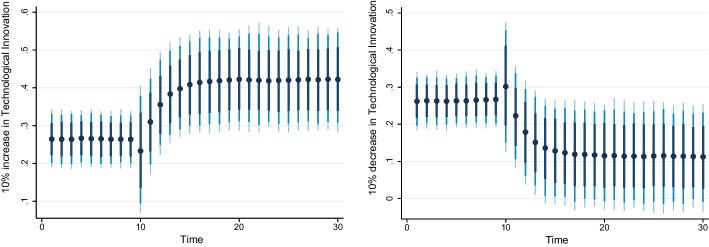
The Impulse Response Plot for Technological Innovation and CO_2_ Emissions. Figure 8 presents an increase and a decrease by 10% in technological innovation and its effect on CO_2_ emissions where dots denote average prediction value. The dark blue to light blue line shows 75, 90, and 95% confidence interval, respectively

Figure 8 depicts the impulse response curves for a 10% increase and decrease in TECH. A 10% increase in technical innovation reduces CO_2_ emissions, but a 10% decrease has an adverse effect on the environment. Over time, CO_2_ emissions increase as TECH declines; nevertheless, each 10% increase in TECH causes a flat decrease in CO_2_ emissions. However, this advancement in technical innovation can support the environment because CO_2_ emissions are still rising.

In addition, this study used the FDC test developed by Breitung and Candelon (2006) to investigate the causality among InSE, InTE, InFISD, InTECH, InEC, InFDI, InOPEN, InIGDP, and InCO_2_ in South Africa. Table 9 indicates that InSE, InTE, InFISD, InTECH, InEC, InFDI, InOPEN, and InIGDP Granger cause InCO_2_ in the short, medium, and long term for frequencies ${\omega }_{i}=0.05, {\omega }_{i}=1.50,{\omega }_{i}=2.50.$ Therefore, InSE, InTE, InFISD, InTECH, InEC, InFDI, InOPEN, and InIGDP have a considerable impact on CO_2_ emissions in South Africa in the short, medium, and long term. Our findings are consistent with those of Udeagha and Ngepah (2019), Sohag et al. (2017), and Al Mamun et al. (2014).

**Table 9:** Frequency-domain causality test

Direction of causality	Long-term	Medium-term	Short-term
	${\upomega }_{\mathrm{i}=0.05}$	${\upomega }_{\mathrm{i}=1.50}$	${\upomega }_{\mathrm{i}=2.50}$
InSE → InCO_2_	< 8.31 >	< 8.50 >	< 9.96 >
	(0.02)**	(0.00)***	(0.00)***
InTE → InCO_2_	< 4.89 >	< 6.49 >	< 6.93 >
	(0.07)*	(0.03)**	(0.04)**
InOPEN → InCO_2_	< 8.94 >	< 8.73 >	< 7.28 >
	(0.00)***	(0.00)***	(0.01)**
InEC → InCO_2_	< 5.12 >	< 6.49 >	< 6.73 >
	(0.08)*	(0.04)**	(0.03)**
InFDI → InCO_2_	< 8.20 >	< 8.08 >	< 8.62 >
	(0.01)**	(0.03)**	(0.00)***
InTECH → InCO_2_	< 4.84 >	< 5.14 >	< 7.83 >
	(0.06)*	(0.04)**	(0.02)**
InFISD → InCO_2_	< 4.20 >	< 6.71 >	< 6.01 >
	(0.06)*	(0.05)**	(0.02)**
InIGDP → InCO_2_	< 5.46 >	< 8.82 >	< 8.89 >
	(0.07)*	(0.00)**	(0.00)**

*, ** and *** denote statistical significance at 10%, 5% and 1% levels, respectively

*Source* Authors’ calculations

**Fig. 9:**
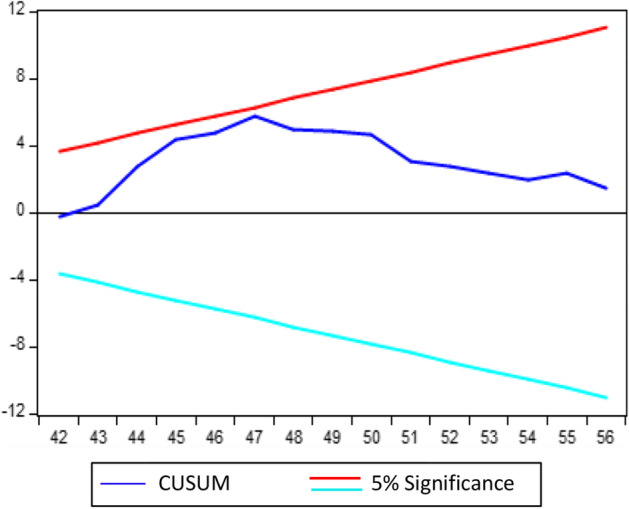
Plot of Cumulative Sum of Recursive Residuals (CUSUM)

**Fig. 10:**
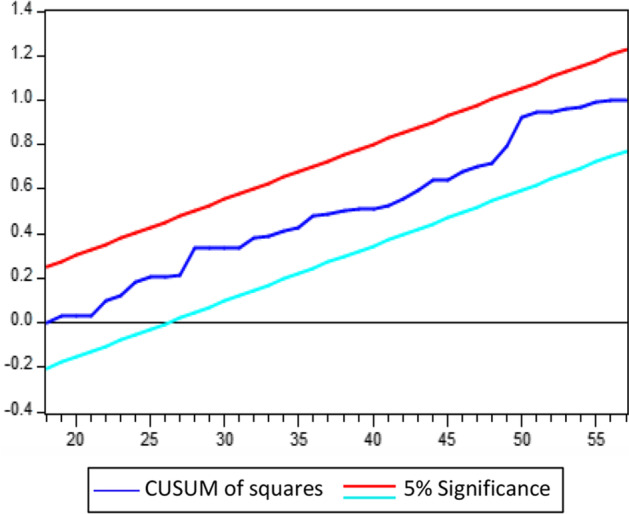
Plot of Cumulative Sum of Squares of Recursive Residuals (CUSUMSQ)

This work study also used the structural performance model evaluation tests to verify robustness. Thus, Pesaran and Pesaran (1997) suggest the CUSUM and CUSUMSQ. Figures 9 and 10 (Appendix) show graphs of CUSUM and CUSUMSQ, respectively. Model parameters are said to be stable over time if plots are under a 5% critical bound threshold. As a result, based on the model trend given in Figs. 9 and 10, we can draw the conclusion that the parameters of the model are consistent and predictable given that CUSUM and CUSUMSQ are inside the bounds at a 5% level.

We accounted for structural breaks as a robustness check as the Gregory–Hansen test of cointegration in Table 10 (Appendix) revealed that the variables are cointegrated in the presence of endogenous breaks. We also created a dummy variable (D1994) to represent the break year of 1994, which corresponded with the end of the apartheid rule and the election of a freely elected government in South Africa. Table 11 presents the results (Appendix), suggesting that the presence of a structural break is not statistically significant.

**Table 10:** Gregory-Hansen test of cointegration with regime shifts: model: change in level

Test	Statistic	Breakpoint	Date	1%	5%	10%
ADF	− 5.40***	40	1994	− 5.01	− 4.51	− 4.30
Zt	− 3.80	37	1996	− 6.19	− 5.39	− 5.30
Za	− 20.95	37	1996	− 53.16	− 25.49	− 22.62
Gregory-Hansen test of cointegration with regime shifts: model: change in level and trend						
ADF	− 5.71**	30	1994	− 5.85	− 5.19	− 4.98
Zt	− 5.05	48	2009	− 5.78	− 5.29	− 5.21
Za	− 25.27	48	2009	− 58.66	− 30.33	− 27.78
Gregory-Hansen test of cointegration with regime shifts: model: change in regime						
ADF	− 5.74**	37	1994	− 6.25	− 5.26	− 5.08
Zt	− 4.75	35	1993	− 6.14	− 5.48	− 5.30
Za	− 36.41	35	1993	− 50.82	− 47.50	− 39.51
Gregory-Hansen test of cointegration with regime shifts: model: change in regime and trend						
ADF	− 5.68*	37	1994	− 6.30	− 5.85	− 5.42
Zt	− 5.34	35	1993	− 6.30	− 5.75	− 5.47
Za	− 30.58	35	1993	− 58.29	− 45.19	− 40.44

*, ** and *** denote statistical significance at 10%, 5% and 1% levels respectively

*Source* Authors’ calculations

**Table 11:** Dynamic ARDL simulations analysis controlling for structural break

Variables	Coefficient	St. Error	t-value
Cons	− 1.1405	1.2870	− 0.93
D94	0.0318	0.1520	0.42
InSE	0.2012***	0.1794	3.04
$\Delta$ InSE	0.3201**	0.2731	2.41
InTE	− 0.6284**	0.8263	− 3.04
$\Delta$ InTE	− 0.7012	0.1404	− 1.41
InFISD	− 0.3605**	0.1281	2.58
$\Delta$ InFISD	− 0.2296***	0.0651	3.56
InTECH	− 0.7051***	0.5883	− 3.25
$\Delta$ InTECH	− 0.2358**	0.0662	− 2.58
InEC	0.2203***	0.1774	3.13
$\Delta$ InEC	0.5685**	0.1732	2.23
InFDI	0.9741	0.0863	1.15
$\Delta$ InFDI	0.2704**	0.2603	2.31
InOPEN	0.1740**	0.0415	2.74
$\Delta$ InOPEN	− 0.3341**	0.0568	− 2.41
InIGDP	0.3518**	0.1551	2.63
$\Delta$ InIGDP	0.5017	0.2341	0.84
ECT(− 1)	− 0.8304***	0.1273	− 3.26
R-squared	0.7885		
Adj R-squared	0.7753		
N	55		
p val of F-sta	0.0000***		
Simulations	1000		

*, ** and *** denote statistical significance at 10%, 5% and 1% levels, respectively

*Source* Authors’ calculations

## Conclusions and policy implications

This study explores the relevance of fiscal decentralization in influencing environmental quality in South Africa from 1960 to 2020. From a theoretical perspective, the study primarily contributes by investigating the existence of race to the top theory. This theory is frequently attributed to a decentralized fiscal structure that enables local authorities to supervise environmentally harmful businesses and thereby shift them outside of their jurisdiction. The fundamental reason is that regional rivalry may have a “race to the top” effect, resulting in tighter environmental rules at greater degrees of fiscal decentralization, which makes any fiscal decentralization beneficial for the environment. The existing body of literature has already produced several useful findings. Although these studies are well supported by references and serve as an inspiration to us, there is still potential for development. We empirically evaluate the impact of fiscal decentralization on environmental quality in this study using the standard EKC methodology to further confirm whether fiscal decentralization causes governments to engage in an environmental “race to the top.” The study further makes an important contribution to scholarship from methodological and empirical viewpoints by using a sophisticated estimation procedure, the novel dynamic ARDL simulation methodology. This procedure is designed to improve the shortcomings of the previous ARDL model to evaluate different model characteristics in the short and long run. The novel dynamic simulated ARDL model aims to simulate, estimate, and dynamically prepare predictions of counterfactual changes in a single regressor and its impact on regressions while keeping the other independent variables constant (Jordan and Philips 2018). This special framework can continuously compute, reproduce, and explore the graphs of the short- and long-term connections between positive and negative variables. Pesaran et al. (2001) developed the basic ARDL, but it can only evaluate long- and short-term linkages among variables. The study’s parameters are all stationary and have a mixed integration order of I(0) and I(1), allowing the use of a single dynamically simulated ARDL model. The variables investigated in this research are timely enough to fulfill the criteria for creating a novel dynamic simulation ARDL model. Using this framework permits to reveal the counterfeit alterations in the regressors and their impacts on regression. The following are the important conclusions: First, more fiscal decentralization could aid environmental sustainability in the long and short run, revealing a green paradox in the instance of South Africa. The reason may be that the country’s environmental quality improved owing to major local environmental requirements and increased authority of the lower levels of government. South Africa has increasingly devised initiatives to enhance ecological sustainability by empowering the lower levels of the government. Fiscal decentralization is required for South Africa to meet its low CO_2_ emission objectives as there is enough proof of the “race to the top” strategy. This event enables the government to enhance ecological sustainability by implementing a “beggar-thy-neighbor” approach to move environmentally damaging activities to neighboring nations. Second, TECH strengthened the sustainability of the environment in the short and long term. Third, although the scale effect reduced ecological sustainability, the TE enhanced it, affirming the EKC hypothesis. Fourth, energy usage, trade openness, industrial value-added, and FDI eroded ecological quality. Finally, the scale effect, TE, fiscal decentralization, TECH, EC, trade openness, industrial value-added, and FDI Granger cause CO_2_ emissions in the short, medium, and long run, implying that these factors are critical in determining environmental quality.

Based on our research findings, the following policy recommendations are proposed: First, as fiscal decentralization is essential to promote ecological quality, South Africa should adopt policies to enhance environmental sustainability by empowering a lower layer of government, including clarification of duties at the national and local levels, in efforts to accomplish the energy-saving roles of fiscal expenditures. Municipal authorities have better knowledge of emission levels in the manufacturing operations of companies than the national government. Providing local authorities with increased fiscal autonomy can help them invest in ecological sustainability more efficiently. Municipal authorities can raise the inclination of spending to ecological stewardship enhancement as their financial autonomy grows. Furthermore, a large and diverse incentive system could be enhanced by prioritizing ecological management performance, such as ecological initiatives and green GDP, in the authorities’ performance appraisal framework, thereby encouraging authorities to strategically control urban ecological degradation.

Second, FDI regulations should be integrated into the ecological regulatory framework because FDI has the potential to degrade ecological integrity. Such policy implementation can be supported by enacting strict pollution regulations for multinational businesses to prevent South Africa from being a pollution haven for multinational companies and safeguard the nation’s ecological integrity. Meanwhile, the FDI entry requirements and assessment methods should be modified. Moreover, a more thorough evaluation process that is ecologically sound, cleaner, and focused on research and development activities should be implemented (Udeagha and Breitenbach 2023a,b). The government needs to improve its oversight of FDI in environmentally damaging enterprises that contribute to global warming. Greater emphasis should be devoted to the sustainability of FDI utilization while ensuring accessibility, particularly in certain poorly developed areas looking to gain quick economic progress and development via FDI.

Third, as energy utilization degrades ecological quality, South Africa’s government should make it easier for businesses to use energy-saving strategies in their production activities by giving low-interest financial support and promoting the growth and development of firms producing energy-saving appliances as a supportive strategy. Moreover, tax incentives and nonprice initiatives which have no impact on conventional energy costs should be implemented to boost energy efficiency. Funding, fiscal incentives, and tax concessions should be provided to sustainable green forms of energy to move the energy structure away from fossil fuels (Udeagha and Ngepah 2022e). In this context, policymakers should consider additional strategies to guarantee policy implementation that enable the transition from nonrenewable to renewable energy sources to promote efficiency in production processes and improve environmental quality in South Africa. Different energy sources should be prioritized to gain a competitive advantage over nonrenewable forms of energy. Innovative strategies in energy storage should really be considered as a crucial strategic instrument, and they should be managed in tandem with green energy schemes. Furthermore, raising a better understanding of the strategic importance of energy innovations in mitigating climate change is critical. Energy plans should be based on energy innovation to reduce the spillover effects of conventional energy sources.

Fourth, the influence of trade openness and GDP on pollution revealed that South Africa has been offering high-emissions-embedded commodities. Consequently, increasing the larger portion of tradable sustainable products in overall trade is suggested. In light of increased liberalization, the industrial architecture should be modified to incorporate more ecologically friendly and efficient manufacturing techniques (Udeagha and Ngepah 2023). More critically, a multilateral partnership on reducing GHG emissions is required to confront rising cross-border pollution and certain potential consequences. In this context, the South African government should endeavor to build significant international links, particularly to exchange TECHs and minimize emissions. Furthermore, governments should include emission reduction chapters in their trade agreement strategy to assist a shift to ecologically sound sectors and a low-carbon economy that promotes green products and services growth.

Although the current research offers relevant empirical findings and policy recommendations for South Africa, one of the work’s primary limitations is its concentration on CO_2_ emissions as the primary measure of ecological quality. Further study is suggested to have a clear grasp of different indicators for ecological quality, including organic water contaminants, nitrogen oxide emissions, sulfur dioxide emissions, and ecological footprint. Furthermore, our observations of the South African economy are constrained to a specific period. Further work is needed to know better how fiscal decentralization could influence emissions, utilizing an alternative measure for energy usage, such as nonrenewable energy. Finally, as our selection is limited to South Africa, future studies could investigate the influence of fiscal decentralization on emissions in other countries, such as Nigeria or Ghana, to determine if the findings are representative of a larger trend.

## Data Availability

The data relevant to this research is publicly available from the World Development Indicators or obtained from the authors by making a reasonable request.

## Appendix

See Figs. 9, 10 and Tables 10, 11.

## Abbreviations

CO_2_ emissions: Carbon dioxide emissions
EKC: Environmental Kuznets curve
GHGs: Greenhouse gases
MIG: Municipal infrastructure grant
OECD: Organisation for Economic Co-operation and Development
ARDL: Autoregressive distributed lag
FDC: Frequency domain causality
GDP: Gross domestic product
MMQR: Method of moments quantile regression
LMDI: Logarithmic mean Divisia index
IPAT: Impact, population, affluence, and technology
ImPACT: Impact, population, affluence, consumption per unit of GDP, and technology
STIRPAT: Stochastic impacts by regression on population, affluence, and technology
TI: Trade intensity
CTI: Composite trade intensity
OPEN: Trade openness
IGDP: Industrial value-added to GDP
TECH: Technological innovation
EC: Energy consumption
FDI: Foreign direct investment
X: Export
M: Import
KPSS: Kwiatkowski–Phillips–Schmidt–Shin
ADF: Augmented Dickey-Fuller
PP: Phillips–Perron
DF-GLS: Dickey-Fuller GLS
FISD: Fiscal decentralization
SE: Scale effect
TE: Technique effect
CUSUM: Cumulative sum of recursive residuals
CUSUMSQ: Cumulative sum of squares of recursive residuals
ECT: Error correction term
US: United States
AIC: Akaike information criterion
HQIC: Hannan–Quinn information criterion
SIC: Schwarz information criterion
SADC: Southern African Development Community
MENA: Middle East and North Africa
UK: United Kingdom
BEM: Big emerging market
EU: European Union
BRICS: Brazil, Russia, India, China, South Africa
BRI: Belt and road initiative
PHH: Pollution haven hypothesis
ICT: Information and communication technology
WTO: World Trade Organization

## Footnotes

1 Organisation for Economic Co-operation and Development.

2 IPAT: Impact, Population, Affluence, and Technology (Holdren 2018); ImPACT: Impact, Population, Affluence, Consumption per unit of GDP, and Technology (Waggoner and Ausubel 2002; York et al. (2003); STIRPAT: Stochastic Impacts by Regression on Population, Affluence, and Technology (Udeagha and Ngepah 2022c; York et al. (2003; Rosa and Dietz 1998).
